# Comparative transcriptomic analysis reveal genes involved in the pathogenicity increase of *Streptococcus suis* epidemic strains

**DOI:** 10.1080/21505594.2022.2116160

**Published:** 2022-08-28

**Authors:** Jianping Wang, Pujun Liang, Hui Sun, Zongfu Wu, Marcelo Gottschalk, Kexin Qi, Han Zheng

**Affiliations:** a State Key Laboratory of Infectious Disease Prevention and Control, National Institute for Communicable Disease Control and Prevention, Chinese Center for Disease Control and Prevention, Changping, Beijing, China; b OIE Reference Lab for Swine Streptococcosis, MOE Joint International Research Laboratory of Animal Health and Food Safety, College of Veterinary Medicine, Nanjing Agricultural University, Nanjing, China; c Swine and Poultry Infectious Diseases Research Center, Faculty of Veterinary Medicine, University of Montreal, Saint-Hyacinthe, Quebec, Canada; d Department of Clinical Laboratory, Nanxishan Hospital of Guangxi Zhuang Autonomous Region, Guilin, Guangxi Zhuang Autonomous Region, China

**Keywords:** *Streptococcus suis*, epidemic strains, pathogenicity increase, comparative transcriptome, methionine, surface proteins

## Abstract

*Streptococcus suis* epidemic strains were responsible for two outbreaks in China and possessed increased pathogenicity which was featured prominently by inducing an excessive inflammatory response at the early phase of infection. To discover the critical genes responsible for the pathogenicity increase of *S. suis* epidemic strains, the genome-wide transcriptional profiles of epidemic strain SC84 were investigated at the early phase of interaction with BV2 cells. The overall low expression levels of 89K pathogenicity island (PAI) and 129 known virulence genes in the SC84 interaction groups indicated that its pathogenicity increase should be attributed to novel mechanisms. Using highly pathogenic strain P1/7 and intermediately pathogenic strain 89–1591 as controls, 11 pathogenicity increase crucial genes (PICGs) and 38 pathogenicity increase-related genes (PIRGs) were identified in the SC84 incubation groups. The PICGs encoded proteins related to the methionine biosynthesis/uptake pathway and played critical roles in the pathogenicity increase of epidemic strains. A high proportion of PIRGs encoded surface proteins related to host cell adherence and immune escape, which may be conducive to the pathogenicity increase of epidemic strains by rapidly initiating infection. The fact that none of PICGs and PIRGs belonged to epidemic strain-specific gene indicated that the pathogenicity increase of epidemic strain may be determined by the expression level of genes, rather than the presence of them. Our results deepened the understanding on the mechanism of the pathogenicity increase of *S. suis* epidemic strains and provided novel approaches to control the life-threatening infections of *S. suis* epidemic strains.

## Introduction

*Streptococcus suis* (*S. suis*) is an important zoonotic pathogen which can cause meningitis, septicemia, endocarditis, arthritis, pneumonia, and peritonitis in pigs and humans [1]. Serotype 2 and sequence type (ST) 1 are the most frequently reported strains in both infected humans and pigs [1,2]. *S. suis* epidemic strains (serotype 2) were responsible for the outbreaks in 1998 and 2005 in China, characterized by the Streptococcal toxic-shock-like syndrome (STSLS) with high mortality [3,4]. In our previous study, the pathogenicity of three STs in serotype 2 strains was well-characterized and nominated as epidemic ST7, highly pathogenic ST1, and intermediately pathogenic ST25 strains, respectively [5]. Compared with highly pathogenic ST1 and intermediately pathogenic ST25 strains, the pathogenicity of the epidemic strain increased and was featured prominently by inducing an excessive inflammatory response at the early phase of infection [5]. Clarifying the mechanism of pathogenicity increase of epidemic strains is urgently needed to develop effective control and treatment strategies. To date, over 100 virulence genes have been reported to be involved in the pathogenesis of *S. suis* virulent strains [6–10]. They were classified into four categories based on their properties and functions, including surface/secreted elements, enzymes/proteases, transcription factors/regulatory systems, and transporters/secretion systems [7]. Moreover, novel virulence genes were continually identified in *S. suis* strains. However, the crucial genes responsible for the pathogenicity increase of the epidemic strains remain unclear.

The interaction with host cells is crucial in the pathogenesis of *S. suis* strains. Considering the interdependence of various virulence genes during the interaction, the analysis of the genome-wide transcriptional profiles rather than individual genes at specific time points post interaction with host cells could be a valuable strategy to link gene expression to the pathogenic phenotype of *S. suis* strains. In a previous study, microarray analysis was performed to identify the transcriptional profiles of a highly virulent *S. suis* strain at different infection sites *in vivo* infection [11]. The regulated genes were mainly related to transcriptional regulation, metabolism, nutrient acquisition, stress defences, and virulence [11]. However, the information on the regulated genes of *S. suis* strains post-interaction with host cells is still limited. Previous transcriptome studies mainly focused on the responses of host cells (such as THP-1 monocytes, porcine alveolar macrophages, and primary porcine choroid plexus epithelial cells *et al*) infected with *S. suis* strains. The genes of host cells involved in the inflammatory response, host defence, apoptosis, and signal transduction pathways were upregulated, while the genes concerned with cellular proliferation and metabolic process were downregulated [12–15]. The innate immune response plays critical roles in the excessive production of pro-inflammatory cytokine and chemokine induced by *S. suis* epidemic strains [16]. Microglia are the macrophage-like population responsible for the innate immune defence in the brain. The BV2 cells, as cell line that exhibits morphological and functional characteristics of microglia, play important roles in the CNS innate immune inflammation triggered by *S. suis* infection [17]. Our previous study demonstrated that epidemic strains possessed a higher ability to elevate the phosphorylation of MAPK pathway proteins in immortal microglia cell BV2 than ST1 strains did at 4 h post-infection, leading to sequential active DNA binding activity of several transcription factors and then excessive pro-inflammatory cytokine release [18]. Clarifying the transcriptome characteristics of epidemic strain before 4 h post-interaction with BV2 cells was important to elucidate its mechanisms to induce the excessive inflammatory response of BV2 cells. In the present study, we compared the genome-wide transcriptional profile of epidemic strain SC84 to those of highly pathogenic ST1 strain P1/7 and intermediately pathogenic ST25 strain 89–1591 at 2 h and 4 h-post interaction with BV2 cells using next-generation sequencing (RNA-seq). Among three interaction groups at the same time points, the upregulated genes of SC84 interaction groups with the highest transcription level were identified. The contributions of the identified genes to the pathogenesis of epidemic strains were further evaluated.

## Materials and methods

### Culture of BV2 cells and *S. suis* strains

BV2 cells were purchased from the Cell Resource Center, IBMS, CAMS/PUMC (Beijing, China). The cells were cultured in Dulbecco’s minimal essential medium (DMEM) with high glucose (Gibco, Carlsbad, USA) supplemented with 10% heat-inactivated foetal bovine serum (Gibco). For each experiment, 3 × 10^5^ cells per well was plated into a 24-well flat-bottomed plates (Becton Dickinson and Company, Minneapolis, USA) and maintained at 5% CO_2_ 37°C for 48 h to allow the cells to grow to confluency (1.5 × 10^6^ cells per well) before the interaction with *S. suis* strains. The medium was changed every 24 h.

*S. suis* strain SC84 (the complete genome accession no. FM252031) as representative of epidemic strains, P1/7 (the complete genome accession no. AM946016) as representative of highly pathogenic strains, and 89–1591 as representative of intermediately pathogenic strains were used in the present study. The complete genome of 89–1591 was sequenced using PacBio Sequel platform and Illumina NovaSeq PE150 in the present study. The *S. suis* strains were grown overnight on Columbia blood base agar plates (Oxoid, London, UK) at 5% CO_2_ 37°C, and isolated colonies were inoculated into 5 ml Todd – Hewitt broth (THB; Oxoid) without agitation at 5% CO_2_ 37°C until OD600 nm value reaching 0.6 in the logarithmic growth phase, which corresponded to 1 × 10^9^ CFU/ml, 1 × 10^9^ CFU/ml, and 5 × 10^8^ CFU/ml for SC84, P1/7, and 89–1591, respectively. The strains were washed twice in PBS (pH 7.4; Gibco). The pellets were then resuspended in DMEM with high glucose medium to obtain an expected concentration of 1 × 10^7^ CFU/ml for each strain. Serial dilutions of the suspension were plated onto THB agar plates to determine the final number of CFU for each experiment.

### Interaction with BV2 microglial cells

Bacterial suspensions (1 ml per well, 72 wells per strain per time point) were added to BV2 cells (MOI was about 7 for each strain). The plates were centrifuged at 500 *g* for 10 min and incubated at 5% CO_2_ 37°C for 0, 2 and 4 h. The 0 h post-interaction groups were used as blank for each strain. Experiments were biologically repeated at least three times for each strain at each time point.

#### Enrichment of bacteria RNA and depletion of rRNA

After the interaction, the medium of each well was removed, and bacterial cells in each well were collected in 1 ml PBS by repeated washing 10 times. The strain suspension was filtered by 5 μm minisart syringe filter (Sartorius, Gottingen, German) twice to remove the BV2 cells, then washed twice in PBS. The total RNA of the pallet was extracted using the TRIzol (Thermo Fisher Scientific, Loughborough, U.K.) according to the manufacturer’s instructions. The MICROB Express Kit (Ambion, Austin, USA) and MICROB Enrich *Kit* (Ambion) were used to enrich bacterial mRNAs in total RNA samples by removing rRNAs and polyadenylated mRNAs according to the manufacturer’s instructions. Prior to sequencing, RNA samples were further quantified and examined for protein and reagent contamination using a Nanodrop ND‐1000 spectrophotometer. The integrity of RNA was determined by running the RNA sample on an Agilent Bioanalyzer RNA 6000 Nano/Pico Chip. The 100 ng purified total RNA samples with RIN (RNA Integrity Number) exceeded seven were used to construct RNA-Seq libraries. Totally, 34 samples (12 for the SC84 interaction and blank groups, 13 for the P1/7 interaction and blank groups, and nine for the 89–1591 interaction and blank groups) were obtained (Supplemental Table 1).

**Table 1.** The information of PICGs identified in the SC84 interaction groups.

		Corresponding homologous gene in P1-7 and 89-1591 genomes				
Post-interaction	PICGs identified in the SC84 interaction groups	P1-7	89-1591	Annotation of PICGs	NO. of UDC	Subgroup of COGs
2 h	SSUSC84_1405	SSU1375	MUN40_07820	Cystathionine β- lyase	UDC14	E
	SSUSC84_1406	SSU1376	MUN40_07825	Cystathionine γ-synthase		E
	SSUSC84_1600	SSU1574	MUN40_02800	Methionine ABC transporter permease	UDC15	E
	SSUSC84_1601	SSU1575	MUN40_02795	Methionine ABC transporter ATP-binding protein，ATPase component		E
	SSUSC84_1602	SSU1576	MUN40_02790	Peptidase		E
	SSUSC84_1603	SSU1577	MUN40_02785	MetQ		E
	SSUSC84_1605	SSU1579	MUN40_02780	5-methyltetrahydropteroyltriglutamate – homocysteine methyltransferase (MetE)	UDC16	E
	SSUSC84_1606	SSU1580	MUN40_02765	5,10-methylenetetrahydrofolate reductase （MetF）		E
	SSUSC84_1835	SSU1813	-	Homocysteine S-methyltransferase（MmuM）	UDC20	E
	SSUSC84_1836	SSU1814	-	Amino acid transporter（MmuP）		E
4 h	SSUSC84_0130	SSU0135	MUN40_01225	Folate family ECF transporter S component	UDC3	E

-: absent in the corresponding genome.

### RNA-Seq sequencing and gene expression analysis

Illumina TruSeq RNA libraries were prepared using the NEB Next Ultra RNA Library Prep Kit (Illumina, San Diego, USA), following the manufacturer’s instructions. cDNA was then ligated to sequencing adapters. After purification and enrichment by PCR, the quality of the cDNA library was assessed on an Agilent Bioanalyzer DNA high-sensitivity chip. The constructed cDNA libraries with fragment lengths of approximately 300 bp were sequenced on an Illumina X-TEN platform (Illumina), sequencing 100 bp length for each read. Quality control of the resulting raw reads was performed using Trimmomatic software (v.0.35). The raw RNA-Seq reads were trimmed and filtered by fastQ software (v.0.21.0). For interaction samples, 1.58 to 8 Gb of >Q20 clean data (MAPQ > 20) were obtained (Supplemental Table 1). The >Q20 clean RNA-Seq reads were uniquely mapped and aligned to ORFs of the corresponding *S. suis* genome by Burrows-Wheeler-Alignment Tool (BWA, v.0.7.17) to produce unigenes. The numbers of reads aligning to ORFs was calculated by cufflinks. The ratio of uniquely aligned reads to >Q20 clean RNA-Seq reads for interaction samples ranged from 12.43% to 92.13%, with average coverage to the corresponding *S. suis* genome from 152◊to 1207◊ (Supplemental Table 1).

For differential expression gene (DEG) analysis, the Fragments Per Kilobase per Million (FPKM) value of each gene was calculated by cufflinks. The FPKM value of each gene in the SC84 interaction groups was compared with that of its homologous gene in the SC84 blank group using the edgeR program in BioConductor [19]. DEGs of the SC84 interaction groups were identified when the *p*-value was＜0.05 and the fold change was＞2. In addition, the FPKM values of genes in the SC84 interaction groups were further compared with these of their homologous genes in the P1/7 and/or 89–1591 interaction groups at the same time point using the edgeR program in BioConductor. The differences in the FPKM values between homologous genes of different groups were significant when the *p*-value was＜0.05 and the fold change was＞2. The biological functions of DEGs were analysed by mapping the clusters of orthologous groups (COG) database by EggNOG V5.0.0 using default parameters (http://eggnogdb.embl.de/#/app/emapper).

The upregulated DEG in the SC84 interaction groups was nominated as pathogenicity increase crucial gene (PICG) if it met one of the following conditions: i. it belonged to epidemic strain-specific gene; ii. its FPKM value was simultaneously higher than that of its homologous gene in the P1/7 and 89–1591 interaction groups at the same time point; iii. its FPKM value was higher than that of its homologous gene in the P1/7 or 89–1591 interaction group at the same time point if its homologous gene was absent in one of genomes. Correspondingly, the non-DEGs in the SC84 interaction groups were nominated as pathogenicity increase-related genes (PIRGs) if they met one of the aforementioned second and third conditions.

To identify the homologous genes among three genomes, a matrix describing the gene content of three genomes was constructed using OrthoMCL. The cut-off value of BLAST E value, identity, and coverage was 1e^5^, 80%, and 80%, respectively. Mobile genes were excluded from three genomes based on the insertion sequence (IS) and transposon databases from EBI and GenBank. Multi-copy genes were also excluded from three genomes.

### Construction of mutants deficient for PICGs of the SC84 2 h post-interaction group

Genomic DNA of SC84 was extracted and purified with the Wizard Genomic DNA Purification kit (Promega, Madison, USA). Restriction enzymes were purchased from New England Biolabs (NEB, Ipswich, USA) and used according to the manufacturer’s recommendations. Corresponding PICGs deletion fragments were constructed using splicing-by-overlap-extension PCR. Primers for the construction of mutants are shown in Supplemental Table 2. The ID5 and ID8 primers of each mutants were spliced with restriction enzyme *Pst*I, *Bam*HI/*EcoR*I restrictive site 6 bp and corresponding protective base 3 or 4 bp at 5’ side (Supplemental Table 2). The PCR amplicons were digested with corresponding double restriction enzymes and subcloned into the thermosensitive *E. coli-S. suis* shuttle vector pSET4s [20] digested with identical double restriction enzymes. The recombinant vectors were transformed into TOP10 competent cells (TIANGEN, Beijing, China) as recommended by the manufacturer. The PICG-deletion constructs were extracted and purified with Plasmid Mini Kit (Omega Bio-Tek, Guangzhou, China), then electroporated into competent *S. suis* strain SC84 using Gene Pulser X cell apparatus (Bio-Rad, Hercules, USA) under specific conditions (2.5 kV/cm, 200Ω, and 25 μF). After electroporation, the competent cells were plated on THA supplemented with Spectinomycin (100 μg/ml, THA + Sp) and incubated for 3 days at 28°C. Several Sp-resistant colonies were then subcultured again on THA + Sp for 2 days at 28°C. The colonies were next cultured on THA + Sp and incubated for 1 days at 37°C for two successive passages and then screened for the first crossing-over event. Loss of vector was induced by incubation of candidates at 28°C at least 10 successive passages. Temperature- and Sp-resistant clones were successively cultured on THA and THA + Sp at 37°C to obtain Sp-sensitive candidates. Deletion of the genes was confirmed by PCR and sequencing analysis. The draft genomes of constructed mutants were sequenced by Illumina sequencing to ensure that the sequences between the wild-type strain and mutants were identical, except for the PICGs deletion.

**Table 2.** The information of PIRGs identified in the SC84 interaction groups.

		Corresponding homologous gene in P1-7 and 89-1591 genomes				
Post-interaction	PICGs identified in SC84 interaction groups	P1-7	89-1591	Annotation of PICGs	NO. of UDC	Subgroup of COGs
2 h	SSUSC84_0164	SSU0171	-	**Epf^d^**		D
	SSUSC84_0178	SSU0186	-	**HP197^d^**		D
	SSUSC84_0242	SSU0253	-	**HP272^d^**		D
	SSUSC84_0314	SSU0327	-	Deoxyguanosinetriphosphatetriphosphohydrolase		F
	SSUSC84_0342	SSU0356	MUN40_00410	Endonuclease		L
	SSUSC84_0347	SSU0361	MUN40_00435	DNA-directed RNA polymerase subunit delta	C1	H
	SSUSC84_0348	SSU0362	MUN40_00440	Substrate-specific component MtsA of methionine-regulated ECF transporter		E
	SSUSC84_0349	SSU0363	MUN40_00445	Hypothetical protein		S
	SSUSC84_0512	SSU0528	MUN40_09220	**Acyltransferase^e^**		M
	SSUSC84_0619	SSU0652	-	Type I restriction modification protein	C3	V
	SSUSC84_0620	SSU0653	-	Type I restriction modification protein		V
	SSUSC84_0621	SSU0654	-	Restriction endonuclease subunit S		V
	SSUSC84_0622	SSU0655	-	Hypothetical protein		S
	SSUSC84_0688	SSU0724	MUN40_04630	Membrane protein		S
	SSUSC84_0772	SSU0809	-	Hypothetical protein		S
	SSUSC84_0777	SSU0814	-	Hypothetical protein	C4	S
	SSUSC84_0778	SSU0815	-	Hypothetical protein		S
	SSUSC84_0932	SSU0887	MUN40_05795	O-acetylhomoserine (thiol)-lyase		E
	SSUSC84_0985	SSU0945	MUN40_06185	**CiaR^d^**		T
	SSUSC84_1029	SSU0991	MUN40_06405	Membrane protein	C5	S
	SSUSC84_1030	SSU0992	MUN40_06410	Transporter		P
	SSUSC84_1333	SSU1303	MUN40_07265	Lipoprotein		S
	SSUSC84_1375	SSU1345	-	Transcriptional regulator	C7	K
	SSUSC84_1376	SSU1346	-	Hypothetical protein		S
	SSUSC84_1664	SSU1639	MUN40_02365	Lipoprotein		G
4 h	SSUSC84_0145	SSU0152	MUN40_01300	**Sspep^d^**		O
	SSUSC84_0242	SSU0253	-	**HP272^d^**		D
	SSUSC84_0249	SSU0260	MUN40_01895	Alcohol dehydrogenase		C
	SSUSC84_0440	SSU0456	MUN40_08175	Membrane protein		S
	SSUSC84_0566	SSU0593	MUN40_05180	Hypothetical protein	C2	S
	SSUSC84_0567	SSU0594	MUN40_05175	Hypothetical protein		S
	SSUSC84_0568	SSU0595	MUN40_05170	Hypothetical protein		S
	SSUSC84_0773	SSU0810	-	Transcriptional regulator		K
	SSUSC84_1072	SSU1034	MUN40_06625	ArsR family transcriptional regulator	C6	K
	SSUSC84_1073	SSU1035	MUN40_06630	MFS transporter		E
	SSUSC84_1501	SSU1473	MUN40_09110	Membrane protein	C8	S
	SSUSC84_1502	SSU1474	MUN40_09115	Hypothetical protein		S
	SSUSC84_1662	SSU1637	MUN40_02375	Pyruvate dehydrogenase E1 component subunit alpha		C
	SSUSC84_1906	SSU1886	-	**Major pilussubunitd**		D

-: absent in the corresponding genome.

d: known virulence gene.

e: gene of cps locus.

### Experimental infection

- Survival assay. C57BL/6 mice (6 weeks old, female) were injected intraperitoneally with 1 × 10^7^ CFU of the wild-type strain SC84 and ∆PICGs mutants in 1 mL THB or 1 mL THB only as the control group. Each infected group contained ten mice and the control group contained five mice. The mortality was recorded per 6 h within 24 h post-infection and per 12 h from 24 h to 72 h post-infection. The experiment was performed independently twice. In the zebrafish infection model [21], 15 adult zebraﬁsh per group were intraperitoneally injected with 3 × 10^5^ CFU and 3 × 10^6^ CFU of wild-type strain SC84 and ∆PICGs mutants in 1 mL THB or 1 mL THB only as the control group. Mortality was monitored until 96 h post-infection. Survival rates of both infection models were calculated via the Kaplan–Meier method.
- Cytokine production *in vivo* and bacterial loads in peripheral blood. C57BL/6 mice (6 weeks old, female) were injected intraperitoneally with 1 × 10^6^ CFU of the wild-type strain SC84 and ∆PICGs mutants in 1 mL THB. Each group contained five mice. At 8 h post-infection, the mice were killed, and the peripheral blood of each infected mouse was collected aseptically. These experiments were performed independently twice. One hundred microlitres of serial tenfold dilutions of peripheral blood of each infected mouse were plated onto blood agar plates. Colonies were counted and expressed as CFU/ml. All serum samples were tested with concentrations of IL-6 and TNF-α using the ELISA kit (R&D Systems, Minneapolis, USA), as recommended by the manufacturer.

### Statistical analysis

The survival rates of different infected groups were compared using the Log-rank test. Statistical analyses of the cytokine data and bacterial counts between wild-type strain SC84 and ∆PICGs mutants infected groups were performed by using the Wilcoxon’s two-sample test. The growth rates of different strains were compared using the Student unpaired *t* test. For these tests, a *p*-value <0.05 was considered to be significant. The statistical analysis was performed using GraphPad Prism software.

### Nucleotide sequence accession number

The sequence of the 89–1591 genome sequenced in the present study was deposited in the GenBank under accession number PRJNA822771. The transcriptome data obtained in the study were deposited in the GenBank under accession numbers listed in Supplemental Table 1.

### Ethical statement

This C57BL/6 mouse infection experiment was approved by the ethics committee of the National Institute for Communicable Disease Control and Prevention, Chinese Center for Disease Control and Prevention (Permit code: 2022–004). This zebrafish infection experiment was approved by the Laboratory Animal Monitoring Committee of Jiangsu Province (Permit code: SYXK (Su) 2017–0007).

## Results

### The homologous and specific genes identified among three genomes

Overall, 1576 genes shared by three genomes were nominated as core genes of three genomes. Among non-core genes, 272 genes only shared by SC84 and P1/7 genomes, 10 genes only shared by SC84 and 89–1591 genomes, and one gene only shared by P1/7 and 89–1591 genomes were identified. In addition, 58, 15, and 456 specific genes were identified in SC84, P1/7, and 89-1591 genomes, respectively (Supplemental Figure 1).

**Figure 1.**
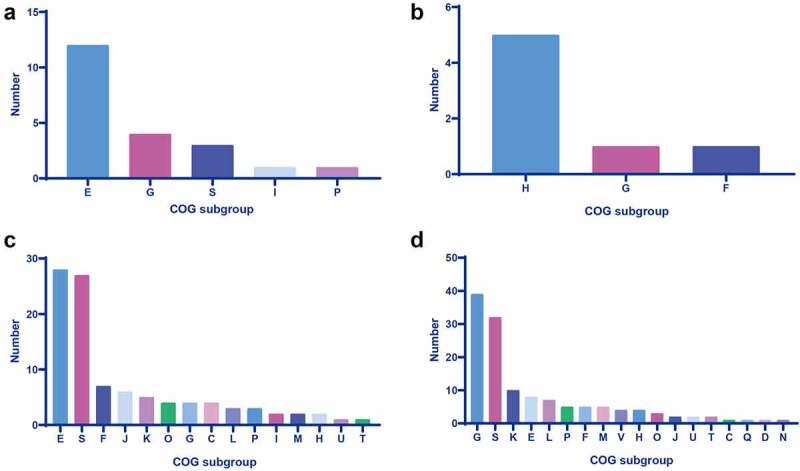
Cog analysis of DEGs in the sc84 interaction groups. A. Upregulated DEGs in the 2 h post-interaction group. B. Downregulated DEGs in the 2 h post-interaction group. C. Upregulated DEGs in the 4 h post-interaction group. D. Downregulated DEGs in the 4 h post-interaction group. The subgroups of cog are indicated in the bars with different colour.

### The DEGs identified in the SC84 interaction groups

In the present study, 28 and 231 DEGs were identified in the SC84 2 h and 4 h post-interaction groups, respectively. Eighteen DEGs were identified in both 2 h and 4 h post-interaction groups, including 13 upregulated DEGs and 5 downregulated DEGs (Supplemental Table S3–1 and 3–2). Totally, 241 DEGs were identified in the SC84 interaction groups. Notably, none of the epidemic strain-specific genes were upregulated in the SC84 interaction groups, while nine epidemic strain-specific genes were downregulated in the SC84 4 h post-interaction group.

*i. 2 h post-interaction group*

Compared with the blank group, a total of 28 DEGs were identified, of which 21 displayed an upregulation and 7 displayed a downregulation. Except for homologous genes of three DEGs *SSUSC84_0921*, *SSUSC84_1835*, and *SSUSC84_1836* absent in the 89–1591 genome, the remaining 25 DEGs belonged to core genes of three genomes. The homologous genes of *SSUSC84_1406*, *SSUSC84_1605*, and *SSUSC84_1606* were also upregulated in both P1/7 and 89–1591 2 h post-interaction groups. The upregulation of the homologous genes of *SSUSC84_0921* and *SSUSC84_0739* was also found in the P1/7 2 h post-interaction group. The homologous genes of *SSUSC84_0446*, *SSUSC84_0674*, *SSUSC84_1603*, and *SSUSC84_1842* also displayed the upregulated transcription level in the 89–1591 2 h post-interaction group. Remaining 12 DEGs were specifically upregulated in the SC84 2 h post-interaction group (Supplemental Table S3–1).

Seven downregulated DEGs were discovered in the SC84 2 h post-interaction group. All of them were the core genes of three genomes. Moreover, their homologous genes were also downregulated in the P1/7 2 h post-interaction group (Supplemental Table S3–2).

In the present study, the biological function of DEGs was investigated by the COG analysis. The COG of upregulated DEGs was predominantly categorized into subgroup of amino acid transport and metabolism, whereas downregulated DEGs mainly encoded coenzyme transport and metabolism-related protein (Supplemental Table S3–1, S3–2, and Figure 1).

*ii. 4 h post-interaction group*

Significantly more DEGs were identified in the SC84 4 h post-interaction group, consisting of 99 upregulated and 132 downregulated DEGs. The majority of the DEGs (196/231) were core genes of three genomes. No homologous genes of seven upregulated and 24 downregulated DEGs were found in the 89–1591 genome, whereas the homologous genes of 13 downregulated DEGs were absent in the P1/7 genome (Supplemental Table S4–1 and S4–2).

Among 99 upregulated DEGs, 28 of them were specifically upregulated in the SC84 4 h post-interaction group, whereas the homologous genes of 29 DEGs also displayed the upregulated transcription level in both P1/7 and 89–1591 4 h post-interaction groups (Supplemental Table S4–1). In addition, the homologous genes of 16 and 26 DEGs were upregulated in the P1/7 and 89–1591 interaction groups at the same time point, respectively. Forty-six DEGs were specifically downregulated in the SC84 4 h post-interaction group. The remaining 86 downregulated DEGs displayed a downregulation in the P1/7 and/or 89–1591 interaction groups at the same time point (Supplemental Table S4–2).

The predominant COG subgroup of 70 upregulated DEGs with known function was amino acid transport and metabolism (n = 28). In contrast, 99 downregulated DEGs with known function were mainly categorized into the COG subgroup of carbohydrate transport and metabolism (n = 39) (Supplemental Table S4–1, S4–2 and Figure 1).

### The clustering distribution of DEGs

About 60% of DEGs (160/241) consisted of two or more contiguous genes in clusters ranging from 0.5 to 7.7 kbp in size. Totally, 53 clusters were identified, among which, 23 upregulated DEG cluster (UDCs) composed of 61 upregulated DEGs and 30 downregulated DEG cluster (DDCs) composed of 99 downregulated DEGs were found (Supplemental Table S3–1, S3–2, S4–1, and S4–2). Five UDCs and two DDCs were present in the SC84 2 h post-interaction group, whereas 20 UDCs and 29 DDCs were present in the SC84 4 h post-interaction group. Among them, UDC14, UDC20, and DDC21 were consecutively regulated in both 2 h and 4 h post-interaction groups (Supplemental Table S3–1 and S4–2).

### Transcriptional activity of 89K PAI, known virulence genes and cps locus in the SC84 interaction groups

None of upregulated DEG was identified among transcriptionally active genes of 89K PAI in either 2 h or 4 h interaction groups, whereas only 13 downregulated 89K PAI genes were found in the 4 h post-interaction group (Supplemental Table S4–2).

In the present study, we also investigated the expression patterns of *S. suis* 129 known virulence genes in the SC84 interaction groups (Supplemental Table S5). Most of these virulence genes (103/129) genes were core genes of three genomes, whereas 25 and 7 genes were absent in the 89–1591 and P1/7 genome, respectively. Six upregulated and nine downregulated virulence genes were identified in the SC84 interaction groups (Supplemental Table S3–1, S4–1, S4–2, and S5). However, the genes encoding the virulence marker preferentially present in highly pathogenic *S. suis* serotype 2 strains, including *mrp*, *sly*, *epf*, *sof*, *nisK*, *nisR*, *salK*, *salR*, *revS*, *SSU05_0473*, *neuB* and *neuC* [22,23], were identified as non-DEGs in the SC84 interaction groups.

Only the *glutamate dehydrogenase* (*gdh*) gene was upregulated in the SC84 2 h post-interaction group (Supplemental Table S3–1 and S5). It was a core gene of three genomes. Interestingly, the gene was subsequently upregulated in the P1/7 and 89–1591 4 h post-interaction groups (data were not shown).

Fourteen virulence genes were identified as DEGs in the SC84 4 h post-interaction group. They were core genes of three genomes, except for *hp197* and *pnuc* absent in the 89–1591 genome. The transcription levels of *hp197*, *grpe*, *arcA*, *cia*H, *cia*R, *SSU05_1311*, *feoB*, *gtfA*, and *snt* genes were downregulated, whereas *38 kDa*, *igA1*, *mutT*, *pnuc*, and *nadR* genes were upregulated (Supplemental Table S4–1, S4–2, and S5).

Low expression levels of *cps* cluster genes were also discovered in the SC84 interaction groups. Four genes were downregulated in the 4 h post-interaction group, and none of the genes were upregulated during the whole interaction (Supplemental Table S4–2).

### The PICGs identified in the SC84 interaction groups

Ten PICGs *SSUSC84_1405*, *SSUSC84_1406*, *SSUSC84_1600*, *SSUSC84_1601*, *SSUSC84_1602*, *SSUSC84_1603*, *SSUSC84_1605*, *SSUSC84_1606*, *SSUSC84_1835*, and *SSUSC84_1836* were identified in the 2 h post-interaction group, while only the *SSUSC84_0130* was identified as PICG in the 4 h post-interaction group (Table 1).

Notably, 10 PICGs of the 2 h interaction group also constituted UDC14, UDC15, UDC16, and UDC20 ranging from 2.3 to 4.1 kb in size, respectively. One PICG of the 4 h post-interaction group constituted UDC3 with an upregulated DEG SSUSC84_0131 (Table 1).

The PICGs *SSUSC84_1405* and *SSUSC84_1406* of UDC14 encoded cystathionine β- lyase and cystathionine γ-synthase, respectively. The function of proteins encoded by four PICGs *SSUSC84_1600*, *SSUSC84_1601*, *SSUSC84_1602*, and *SSUSC84_1603* of UDC15 was methionine ABC transporter permease, methionine ABC transporter ATP-binding protein, peptidase M20, and MetQ, respectively. The UDC16 was composed of two PICGs *SSUSC84_1605* and *SSUSC84_1606* which encoded 5-methyltetrahydropteroyltriglutamate-homocysteine methyltransferase (MetE) and 5,10-methylenetetrahydrofolate reductase (MetF), respectively. UDC20 contained two PICGs *SSUSC84_1835* and *SSUSC84_1836* which encoded homocysteine S-methyltransferase and homocysteine S-methylpermease, respectively. Besides MetE and MetH, homocysteine S-methyltransferase and homocysteine S-methylpermease constituted a third methionine synthase in *E. coli* [24]. The only PICG *SSUSC84_0130* of the SC84 4 h post-interaction group encoded folate family ECF transporter S component. Notably, the COG of all PICGs was categorized into amino acid transport and metabolism-related protein. All of them were related to the pathway of methionine biosynthesis and uptake.

### The PICGs of the 2 h post-interaction group played different roles in pathogenesis of SC84

The role of PICG *SSUSC84_0130* of the 4 h post-interaction group in the pathogenesis of *S. suis* has been well studied [25,26]. Therefore, we focus on the PICGs of the 2 h post-interaction group in the present study. To assess the contribution of PICGs identified in the 2 h post-interaction group to the pathogenicity of SC84, the knock-out mutants deficient for UDC14, UDC15, UDC16, and UDC20 were constructed.

**Figure 2.**
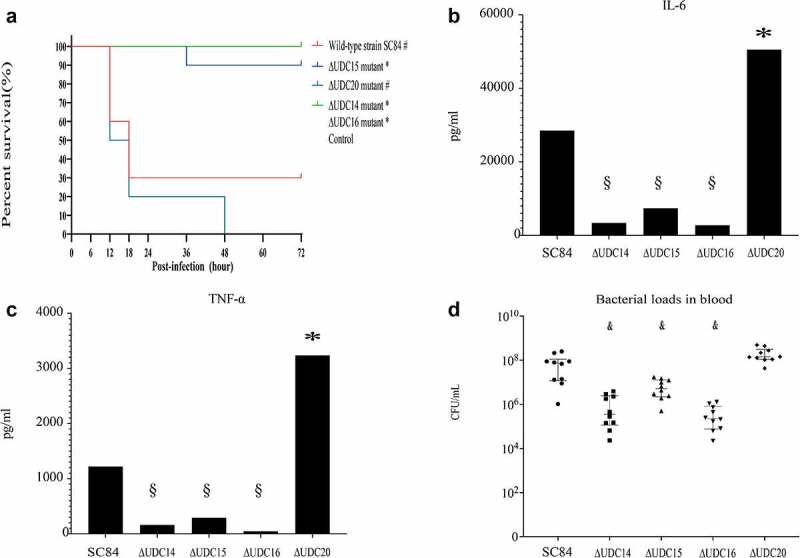
Survival curves of mice injected with 1 × 10^7^ CFU of wild-type strain SC84, △UDC14 mutant, △UDC15 mutant, △UDC16 mutant, △UDC20 mutant, and PBS only as control group (A). Survival rates were calculated via the Kaplan–Meier method. The experiment was performed independently in duplicate. Mean of survival rates were present. The survival rates of different groups were compared using Log-rank test. # significant difference in survival level between the infected group and control group. * significant difference in survival level between the mutant and SC84 infected group. Production of proinflammatory cytokines IL-6 (B) and TNF-α (C) in serum and bacterial loads in peripheral blood (D) of C57BL/6 mice at 8 h post infected with 1 × 10^6^ CFU of wild-type strain SC84 and △udcs mutants. The experiment was repeated twice with similar results. Median of cytokine levels in serum were presented. Peripheral blood bacterial loads were presented in median with interquartile range. Statistical analysis of the cytokine and bacterial counts was performed using the Wilcoxon two-sample test. *p* <0.05 was considered significant. * the cytokine levels were significantly higher than those of mice infected with wild-type strain SC84. § the cytokine levels were significantly lower than those of mice infected with wild-type strain SC84. # the bacterial loads in blood were significantly lower than those of mice infected with wild-type strain SC84.

The wild-type strain SC84 and mutants reached the exponential phase at 3 h post incubation in THB medium. No significant difference in viable counts was observed between SC84 and mutants (Supplemental Figure 2). The comparative pathogenicity analysis was performed in C57BL/6 mouse and zebrafish infection models between mutants and wild-type strains. The survival rate of mice infected with wild-type strain SC84 was 60% at 12 h post-infection, then decreased to 30% at 18 h post-infection. Compared with that of mice infected with wild-type strain SC84, the significantly higher percentage of mice infected with ∆UDC14, ∆UDC15, and ∆UDC16 mutants survived throughout the experiment. The mice infected with ∆UDC14, ∆UDC15, and ∆UDC16 mutants still had a 100%, 90%, and 100% survival rate at 72 h post-infection, respectively. Moreover, the survival rates of mice infected with three mutants were similar to that of mock-infected mice. In contrast, no significant difference in survival rate between the mice infected with ∆UDC20 mutant and wild-type strain SC84 was observed. The survival rate of ∆UDC20 mutant infected mice was 50% at 12 h post-infection, then dramatically decreased to 20% at 24 h post-infection and 0% at 48 h post-infection (Figure 2A).

Excessive proinflammatory cytokines played critical roles in the pathogenesis of epidemic strains [5,16,27]. The capacity of ∆UDC mutants to induce the production of pro-inflammatory cytokines in C57BL/6 mice was compared with that of wild-type strain SC84. Production of proinflammatory cytokines TNF-a and IL-6 in mice infected with ∆UDC20 mutant, was significantly higher than those of mice infected with wild-type strain SC84 at 8 h post-infection (Figure 2 B and C). No significant difference was observed in bacterial loads of the peripheral blood between two infected groups. The bacterial counts in the peripheral blood of both wild-type strain SC84 and ∆UDC20 mutant infected groups reached 10^8^ CFU/mL at 8 h post-infection (Figure 2D). In contrast, the pro-inflammatory cytokine levels in serum and bacterial loads in peripheral blood of the mice infected with ∆UDC14, ∆UDC15, and ∆UDC16 mutants were significantly lower than those of mice infected with wild-type strain SC84 at 8 h post-infection (Figure 2 B and C).

**Figure 3.**
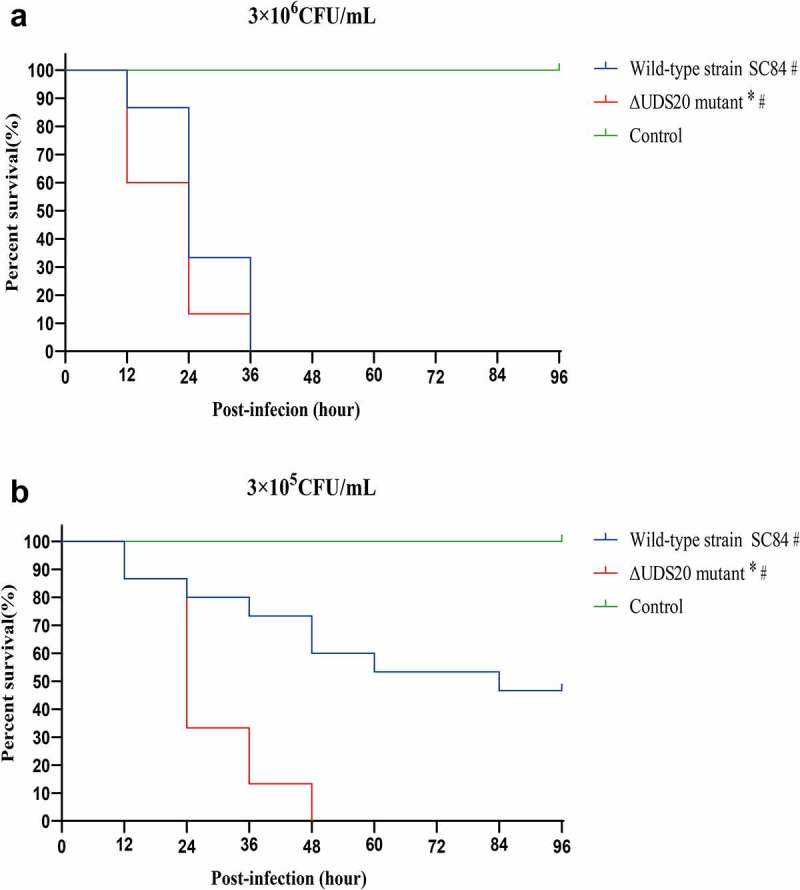
Survival curves of zebrafish infected with 3 × 10^6^ CFU (A) and 3 × 10^5^ CFU (B) of wild-type strain SC84 and △UDC20 mutant. PBS only was used in control group. Survival rates were calculated via the Kaplan–Meier method. The experiment was performed independently in duplicate. Mean of survival rate were presented. The survival rates of different groups were compared using Log-rank test. #, significant difference in survival level between infected group and control group. *, significant difference in survival level between △UDC20 mutant and wild-type strain SC84 infected groups. P < 0.05 was considered significant.

In order to further evaluate the pathogenicity of ∆UDC20 mutant, the zebrafish infection model was also used (Figure 3). The zebrafish as a convenient, reliable, and standardized model was wildly applied to the assess the virulence level of *S. suis* [21,28–30]. In contrast to the result of mouse infection model, the survival rate of ∆UDC20 mutant infected group was significantly lower than that of the wild-type strain SC84 infected groups in the zebrafish infection model at both 10^5^ and 10^6^*cfu* infection doses. At 10^5^*cfu* infection dose, ∆UDC20 mutant had a 100% mortality rate to zebrafish at 48 h post-infection, whereas the mortality rate to zebrafish infected with the wild-type strain SC84 was 40% at the same time point. At 10^6^*cfu* infection dose, the difference in survival level between the zebrafish infected with ∆UDC20 mutant and wild-type strain SC84 was mainly observed at the early phase of infection. Zebrafish infected with ∆UDC20 mutant had a 60% survival rate at 12 h post-infection, while survival rate of zebrafish infected with wild-type strain SC84 was 100% at the same time point. The survival rates of both infected groups dramatically decreased to 0% at 36 h post-infection. These data indicated that ∆UDC20 mutant possessed a higher pathogenicity than wild-type strain SC84 did.

### The PIRGs identified in SC84 interaction groups

Among 1576 core genes of three genomes, 12 and 11 PIRGs were identified in the SC84 2 h and 4 h post-interaction group, respectively. None of them were identified as PIRG in both 2 h and 4 h post-interaction groups. Among 272 genes only shared by the SC84 and P1/7 genomes, 13 and 3 PIRGs were identified in SC84 2 h and 4 h post-interaction group, respectively (Table 2). Amongst, only *hp272* gene was identified as PIRG in both 2 h and 4 h post-interaction groups. Totally, 38 PIRGs were identified in the SC84 interaction groups. It is noteworthy that six of them belonged to known virulence genes, consisting of *S. suis* classical virulence gene *epf*, *hp197*, *hp272*, *ciaR*, *sspep*, and *pili* (Table 2 and Supplemental Table 5).

Interestingly, a high proportion of PIRGs encoded surface/membrane proteins. It indicated that surface/membrane proteins play important roles in the excessive inflammatory response induced by epidemic strains. In addition, PIRG *SSUSC84_0348* and *SSUSC84_0932* genes encoded methionine-regulated ECF transporter and O-acetylhomoserine (thiol)-lyase, respectively. Both of them may be involved in the pathway of methionine metabolism.

The feature of clustering distribution was also present in PIRGs. Twenty contiguous PIRGs constituted eight clusters, five (C1, C3, C4, C5, and C7) and three clusters (C2, C6, and C8) were identified in the 2 h and 4 h post-interaction group, respectively (Table 2).

## Discussion

The pathogenicity increase of epidemic strain is characterized by inducing an excessive host inflammatory response at the early phase of infection [5]. In our previous study, the inflammatory responses of BV2 cells infected with epidemic strain as well as highly pathogenic ST1 strain were compared [18]. The phosphorylation level of the MAPK pathway in BV2 cells infected with epidemic strain was significantly higher than that of cells infected with ST1 strain at 4 h post-interaction. Subsequently, BV2 cells challenged epidemic strain produced significantly higher levels of the pro-inflammatory cytokines than BV2 cells infected with ST1 strain did from 8 h post-infection [18]. Considering the fact that the differentiated response of infected BV2 cells was observed at 4 h post-interaction, the whole-genome transcriptome profiles of epidemic strain SC84 at 2 h and 4 h post-interaction with BV2 cells were analysed and compared with those of ST1 strain P1/7 in the present study. In order to eliminate potential false-positive targets and obtain a stringent list of genes related to the pathogenicity increase of epidemic strain, *S. suis* strain 89–1591 with intermediate virulence was also included in the present study.

The regulation of genes in the SC84 interaction groups was time-dependent, whereas 28 and 231 DEGs were identified in the 2 h and 4 h post-interaction group, respectively. Notably, the distribution of two or more contiguous DEG in clusters was wildly present in DEGs of the SC84 interaction groups. These DEG clusters may participate in the infection of epidemic strain as functional modules. Obvious differences were observed in functional categories between upregulated and downregulated DEGs. The predominant COG subgroup of upregulated DEGs was amino acid transport and metabolism. In contrast, downregulated DEGs mainly encoded proteins involved in carbohydrate transport and metabolism. Nutrient availability is closely linked to the adaptations and virulence of pathogens. Our data suggested that metabolites of amino acid metabolism may be indispensable at the early phase of epidemic strain interaction with host cells. Further metabonomic analysis may provide a novel approach to deepen the understanding of the pathogenesis of epidemic strains.

In the present study, the overall transcription level for genes of 89K PAI was low in the SC84 interaction groups. Similar results were also reported in two epidemic strains purely grown in THB medium [31]. T4SS-like system and two-component signal transduction (TCST) systems located in 89K PAI contributed to the development of STSLS and the production of excessive pro-inflammatory cytokines [32–34]. Our results showed that the expression levels of these systems was not regulated throughout the interaction. It indicated that 89K PAI of epidemic strains might not play critical roles at the early phase of infection. Likewise, low transcriptional activities of *S. suis* known virulence genes were also found in the SC84 interaction groups. These results indicated that the pathogenicity increase of epidemic strains might be attributed to novel mechanisms.

Compared with transcriptome profiles of highly pathogenic ST1 strain P1/7 and intermediately pathogenic ST25 strain 89–1591, 10 and one upregulated genes were identified as PICGs in the SC84 2 h and 4 h post-interaction group, respectively. The fact that none of them belonged to epidemic strain-specific genes suggested the pathogenicity increase of epidemic strain may be determined to a great extent by the expression level of genes, rather than their presence. It is noteworthy that 10 PICGs of 2 h post-interaction group constituted four clusters, consisting of UDC14, UDC15, UDC16, and UDC20.

Both PICGs *SSUSC84_1405* and *SSUSC84_1406* constituting UDC14 are pyridoxal phosphate-dependent enzymes and their homologous genes were involved in the transsulfuration pathway of methionine biosynthesis in *Streptococcus anginosus* [35,36]. The proteins encoded by four PICGs of UDC15 were predicted to be involved in the methionine uptake. The genes *SSUSC84_1600*, *SSUSC84_1601*, *SSUSC84_1602*, and *SSUSC84_1603* encoded methionine ABC transporter permease, methionine ABC transporter ATP-binding protein, peptidase, and MetQ, respectively. The arrangement of UDC15 was similar to *S. pneumoniae metQNP* locus encoding a methionine ABC transporter [37]. Moreover, MetQ and ABC transporter were constituents of the Methionine ABC (ATP binding cassette) Uptake Transporter (MUT) family in *Escherichia coli* and *Bacillus subtilis* [38,39]. MetQ was responsible for methionine delivery to the ABC transporter. The *metQ* gene was known to be relevant to the survival of *S. suis* in blood [11], while a serotype 2 *S. suis* strain knocked out *metQ* gene showed decreased anti-phagocytosis ability in infected RAW264.7 cells [40].

The UDC16 was composed of two PICGs *SSUSC84_1605* and *SSUSC84_1606* which encoded 5-methyltetrahydropteroyltriglutamate-homocysteine methyltransferase (MetE) and 5,10-methylenetetrahydrofolate reductase (MetF), respectively. MetE catalysed the synthesis and transfer of methyl group from 5-methyltetrahydrofolate directly to homocysteine [41,42]. The methyl group transferred by MetE to homocysteine is donated by 5-methyltetrahydrofolate which is synthesized from N5,10-methylene tetrahydrofolate by the catalyzation of MetF [43]. Disruption of *metF* leads to methionine auxotrophy in *Streptomyces lividans* [44].

UDC20 contained two PICGs *SSUSC84_1835* and *SSUSC84_1836* which encoded homocysteine S-methyltransferase and permease, respectively. The homologous gene of *SSUSC84_1835* in *E. coli* was *YagD* gene (renamed *mmuM* gene) that catalyzes the last step in methionine biosynthesis and plays an important role in the metabolism process [45]. The homologous gene of *SSUSC84_1836* in *E. coli* was *ykfD* gene (renamed *mmuP* gene) that was responsible for the transporter of S-methylmethionine and co-transcribed with the *mmuM* gene [24]. The physiological roles of the *mmuM* and *mmuP* products appear to uptake the external S-methylmethionine and convert homocysteine into methionine by transferring the methyl to homocysteine [24], which is catalysed by either of two transmethylase enzymes MetE and MetH in enterobacteria [41].

It is noteworthy that the proteins encoded by 10 PICGs of 2 h interaction group were all involved in the pathway of methionine biosynthesis and uptake. It indicates that the methionine and its metabolites are indispensable at the early phase of epidemic strain interaction with BV2 cells. Methionine, a vital amino acid in bacterial metabolism, acts as the initiator of protein synthesis and elongation. In addition, some methionine derivatives (e.g. S-adenosylmethionine) serve as methyl donors for the biosynthesis of phospholipids and nucleic acids [46].

To further evaluate the combined contribution of PICGs in the pathogenicity increase of epidemic strains, the virulence levels of knock-out mutants deficient for UDC14, UDC15, UDC16, or UDC20 were compared with that of their parental strain SC84 using C57BL/6 mouse and zebrafish infection models. The ∆UDC14, ∆UDC15, and ∆UDC16 mutants showed significantly decreased lethality to C57BL/6 mice and pro-inflammatory cytokine levels in serum, which may mainly attribute to their lower bacterial loads in peripheral blood. The results indicated that the upregulation of three UDC14, UDC15, and UDC16 significantly bolstered the capacity of epidemic strains to resist phagocytosis by the host immunological system and rapidly establish infection.

On the contrary, the pathogenicity of ∆UDC20 mutant was higher than that of its parental strain since it showed a higher capacity to stimulate the production of pro-inflammatory cytokines in C57BL/6 mice than its parental strain did at the early phase of infection. In addition, the mortality rate of zebrafish infected with ∆UDC20 mutant was significantly higher than that of the group infected with the wild-type strain SC84 at the early phase of infection. These results indicated that the overexpression of UDC20 negatively regulated the virulence of the epidemic strain.

The differentiated pathogenic phenotypes of the four UDCs indicated they may play different roles in the biosynthesis and metabolism of methionine. The deletion of UDC14, UDC15, and UDC16 may result in methionine auxotrophy owing to the detriment of methionine biosynthesis and uptake, then the capacity of epidemic strains to survive and disseminate in the bloodstream significantly attenuated. On the contrary, significantly increased pathogenicity was observed in the ∆UDC20 mutant. It is possible that the deletion of UDC20 resulted in the accumulation of its substrates (such as S-methylmethionine and S-adenosylmethionine) or the enhanced production of selenomethionyl protein [47], which may in turn lead to the pathogenicity increase of epidemic strains. The deletion of UDC20 may also result in excessive methionine production by upregulated other methionine synthase at the early phase of infection that significantly bolstered the pathogenicity of ∆UDC20 mutant. So far, the pathway of methionine biosynthesis and metabolism in the *S. suis* are still not well characterized. Further studies are urgently needed to elucidate the pivotal components of methionine cycling and their mechanisms in the pathogenicity increase of epidemic strains.

It is noteworthy that six of 10 PICGs were consistently upregulated in the SC84 4 h post-interaction group, consisting of *SSUSC84_1405*, *SSUSC84_1406*, *SSUSC84_1600*, *SSUSC84_1602*, *SSUSC84_1835*, and *SSUSC84_1836*. They were no longer identified as PICGs in the SC84 4 h post-interaction group in that their homologous genes were upregulated in the 89–1591 and/or P1/7 4 h post-interaction groups. It indicated that the extremely rapid initiation of methionine biosynthesis is crucial for the pathogenicity increase of the epidemic strains.

The only PICG identified in the SC84 4 h post-interaction group was the *SSUSC84_0130* gene. The gene encoded folate family ECF transporter S component. Folate is essential for methionine biosynthesis in Streptomyces, Enterobacteria, and Mycobacterium tuberculosis [44,48]. It is possible that the protein encoded by *SSUSC84_0130* gene was also involved in the methionine biosynthesis of *S. suis* strains. In a previous study, a hypervirulent *S. suis* strain (S735-pCOM1-V10) was generated by transforming a 3-kb fragment containing *orf2* gene from virulent serotype 2 strain 10 into the weakly virulent serotype 2 strain S735 [26]. The expression level and specific nucleotide polymorphisms of the fragment were responsible for the pathogenicity increase of S735-pCOM1-V10 [25]. The sequence of *SSUSC84_0130* was identical to that of *orf2* gene. The homologous gene of *SSUSC84_0130* was also upregulated in the 89–1591 2 h and 4 h post-interaction groups, although its FPKM value was lowest among three interaction groups at the same time point. Compared with the sequence of *SSUSC84_0130* gene, 17 base-pair substitutions were identified in the homologous gene of the 89–1591 genome. In a previous study, the expression level of *orf2* was strongly associated with sequences of the predicted −35 region upstream of its promoter [25]. An SNP present in the corresponding region of the 89–1591 genome may be partially responsible for its low FPKM value in the 89–1591 interaction groups.

In addition to PICGs, 38 PIRGs were identified in the SC84 interaction groups, including two genes related to methionine metabolism and six known virulence genes. In addition, the expression level of *hp272* in epidemic strain 05ZY033 5 h post-inoculate in THB medium were significantly higher than that of P1/7 [31]. Although none of the “classical” *S. suis* virulence markers *mrp*, *sly* and *epf* were upregulated in the SC84 interaction groups, *epf* gene was identified as PIRG in the SC84 2 h post-interaction group. It is noteworthy that *mrp* and *sly* genes were upregulated in the P1/7 4 h post-interaction group (data were not shown), although no differences in FPKM values were observed between P1/7 and SC84 interaction groups at the same time point. Compared with *mrp* and *sly* genes, *epf* gene may play critical role in the pathogenicity increase of epidemic strains. In addition, surface proteins encoded by genes *hP197* [49] and *hP272* [50], secretary/immunogenic protein encoded by gene *sspep* [51], and two-component regulatory system encoded by gene *ciaR* [52] promoted the adherence of the *S. suis* to host cells. *SSUSC84_1906* gene (separated into *Sbp2’* and *Sbp2’’* because of a nonsense mutation) coding a major pilin subunit Sbp2. Pili on the surface of Gram-positive strains played important roles in adherence to and invasion of host cells and biofilm formation. Deletion of *Sbp2’* clearly attenuated the virulence of *S. suis* strain P1/7 in a zebrafish model [53].

Interestingly, six upregulated known virulence genes in the SC84 interaction groups also encoded surface proteins. Among them, *gdh* and *38 kDa* gene was mainly related to amino acid and carbohydrate metabolism, respectively [8,54,55]. *igA1* gene assisted *S. suis* strains to break the host mucosal immune barrier [56], *mutT, pnuc*, and *nadR* genes involved in the biosynthesis and utilization pathway of NAD (Nicotinamide Adenine Dinucleotide) that were responsible for the resistance to oxidative stress and evasion of host immunity phagocytosis during the infection [57–59]. In the present study, high proportion of PIRGs and upregulated known virulence genes encoded surface proteins that were involved in host cell adherence, *in vivo* survival, and immune escape, which were first step for epidemic strains to initiate the infection. The capacity to more rapidly initiate the infection may partially be responsible for the pathogenicity increase of epidemic strains. Consistent with our results, it was previously shown that most of the upregulated genes in epidemic strains grown in THB medium *in vitro* encoded surface-anchored proteins [31]. The roles of many PIRGs in the pathogenesis of epidemic strains are still poorly characterized and worthy of further investigation. Surprisingly, the global expression level of capsular polysaccharide (CPS) conferring antiphagocytic properties to epidemic strains was low. Our data indicated that CPS of epidemic strains might not play critical roles in the stage of initiating infection.

In conclusion, the genome-wide transcriptional profiles of epidemic strain SC84 at the early phase of interaction with BV2 cells were investigated. The overall low expression levels of 89K PAI genes and known virulence genes in the SC84 interaction groups indicated that the pathogenicity increase of epidemic strains should be attributed to novel mechanisms. Using highly pathogenic strain P1/7 and intermediately pathogenic strain 89–1591 as controls, we revealed that methionine biosynthesis/uptake pathway played critical roles in the pathogenicity increase of epidemic strains. The proteins related to host cell adherence and immune escape may contribute to the extremely rapid initiation of the infection which is conducive greatly to the pathogenicity increase of epidemic strains. Our study deepened the understanding of the pathogenesis of epidemic strains, meanwhile provided novel approach and valuable information to control the infection of epidemic strains.

## Supplementary Material

Supplemental Material

## Data Availability

The sequence of the genome sequenced in the study was deposited in the GenBank under accession number PRJNA822771. The transcriptome data obtained in the study were deposited in the GenBank under accession numbers SRR18778325-SRR18778333, SRR18778712 -SRR18778724, and SRR18779406-SRR18779417. The data supporting the results presented in the study are available from the corresponding author upon reasonable request.
