# Supplementary material for: Comparative transcriptomic analysis reveal genes involved in the pathogenicity increase of Streptococcus suis epidemic strains
Source: Virulence. 2022 Aug 28;13(1):1455–70. doi: 10.1080/21505594.2022.2116160 (PMC9423846; doi:10.1080/21505594.2022.2116160)
Supplement: Supplemental Material [file KVIR_A_2116160_SM1757.zip › supplementary/Supplemental Tables.docx]

Supplemental Table 1. The information of the transcriptome data obtained in the study and corresponding S. suis genome mapping statistics

| **Sample name** | **Raw data (Gb)** | **>Q20 Clean data (Gb)** | **The ratio of >Q20 Clean data to raw data (%)** | **The ratio of uniquely aligned ORF data to >Q20 Clean data (%)** | **The average coverage of uniquely aligned ORF data to corresponding *S. suis* genome (x)** | **Accession No.** |
| --- | --- | --- | --- | --- | --- | --- |
| SC84-Blank-Ind17 | 3.34 | 3.08 | 92.21 | 97.19 | 1497 | SRR18779416 |
| SC84-Blank-Ind31 | 6.99 | 6.89 | 98.57 | 57.98 | 1997 | SRR18779409 |
| SC84-Blank-Ind38 | 6.98 | 6.9 | 98.85 | 65.56 | 2262 | SRR18779407 |
| SC84-2h-Ind19 | 2.44 | 2.2 | 90.16 | 92.13 | 1013 | SRR18779412 |
| SC84-2h-Ind29 | 4.36 | 4.24 | 97.25 | 19.75 | 419 | SRR18779411 |
| SC84-2h-Ind30 | 3.77 | 3.65 | 96.82 | 25.16 | 459 | SRR18779410 |
| SC84-2h-Ind39 | 5.33 | 5.25 | 98.50 | 13.80 | 362 | SRR18779415 |
| SC84-4h-Ind17 | 2.68 | 2.42 | 90.30 | 66.00 | 799 | SRR18779413 |
| SC84-4h-Ind24 | 2.99 | 2.74 | 91.64 | 87.49 | 1199 | SRR18779417 |
| SC84-4h-Ind31 | 6.32 | 6.23 | 98.58 | 22.87 | 712 | SRR18779408 |
| SC84-4h-Ind38 | 6.7 | 6.6 | 98.51 | 24.36 | 804 | SRR18779406 |
| SC84-4h-Ind43 | 4.69 | 2.62 | 55.86 | 83.26 | 1091 | SRR18779414 |
| P1/7-Blank-Ind18 | 3.04 | 2.8 | 92.11 | 97.46 | 1364 | SRR18778724 |
| P1/7-Blank-Ind32 | 8.1 | 8 | 98.77 | 52.18 | 2087 | SRR18778723 |
| P1/7-Blank-Ind40 | 4.07 | 2.27 | 55.77 | 99.61 | 1131 | SRR18778719 |
| P1/7-2h-Ind20 | 2.8 | 2.5 | 89.29 | 57.30 | 716 | SRR18778718 |
| P1/7-2h-Ind23 | 2.87 | 2.58 | 89.90 | 66.39 | 856 | SRR18778717 |
| P1/7-2h-Ind33 | 5.2 | 5.12 | 98.46 | 12.43 | 318 | SRR18778716 |
| P1/7-2h-Ind41 | 3.53 | 1.73 | 49.01 | 17.61 | 152 | SRR18778715 |
| P1/7-2h-Ind42 | 4.6 | 2.64 | 57.39 | 91.43 | 1207 | SRR18778714 |
| P1/7-4h-Ind21 | 2.52 | 2.29 | 90.87 | 78.33 | 897 | SRR18778713 |
| P1/7-4h-Ind32 | 6.25 | 6.15 | 98.40 | 25.66 | 789 | SRR18778712 |
| P1/7-4h-Ind33 | 5.46 | 5.35 | 97.99 | 26.32 | 704 | SRR18778722 |
| P1/7-4h-Ind35 | 5.05 | 4.98 | 98.61 | 13.49 | 336 | SRR18778721 |
| P1/7-4h-Ind39 | 6.87 | 6.78 | 98.69 | 31.74 | 1076 | SRR18778720 |
| 89-1591-Blank-Ind28 | 2.53 | 2.32 | 91.70 | 96.77 | 1123 | SRR18778333 |
| 89-1591-Blank-Ind34 | 7.17 | 7.08 | 98.74 | 37.45 | 1326 | SRR18778332 |
| 89-1591-Blank-Ind40 | 4.81 | 2.85 | 59.25 | 99.46 | 1417 | SRR18778331 |
| 89-1591-2h-Ind26 | 2.28 | 2.1 | 92.11 | 81.95 | 860 | SRR18778330 |
| 89-1591-2h-Ind27 | 1.71 | 1.58 | 92.40 | 91.84 | 726 | SRR18778329 |
| 89-1591-2h-Ind36 | 4.49 | 4.41 | 98.22 | 15.51 | 342 | SRR18778328 |
| 89-1591-4h-Ind26 | 1.89 | 1.73 | 91.53 | 86.84 | 751 | SRR18778327 |
| 89-1591-4h-Ind28 | 2.76 | 2.53 | 91.67 | 86.42 | 1093 | SRR18778326 |
| 89-1591-4h-Ind34 | 5.33 | 5.24 | 98.31 | 36.05 | 945 | SRR18778325 |

Supplemental Table 2. The information of primers for the construction of △UDC14, △UDC15, △UDC16, and △UDC20 mutants

| **Name** | **Position in SC84 genome** | **Sequence 5'-3'** | **Restriction enzyme** | **Annealing temperature (℃)** | **Size of production (bp)** |
| --- | --- | --- | --- | --- | --- |
| △UDC14-ID1 | 1490213..1490230 | CCGTGGACCTATGTATGG |  | 49 | 1504 |
| △UDC14-ID2 | 1491716..1491699 | GGAGTTGTTCCTGCCTTA |  |  |  |
|  |  |  |  |  |  |
| △UDC14-ID3 | 1492077..1492094 | TGTCAAACCGTAAGAATG |  | 49 | 1562 |
| △UDC14-ID4 | 1493638..1493621 | GTAGGATACAGGCAAGAG |  |  |  |
|  |  |  |  |  |  |
| △UDC14-ID5 | 1490486..1490500 | *CGCGGATCCCAGCAAGCCATCAAG | *Bam*HI | 47 | 565 |
| △UDC14-ID6 | 1491050..1491036 | #AACACCGAGGAGGAGATGAAGCCTCAGGGA |  |  |  |
|  |  |  |  |  |  |
| △UDC14-ID7 | 1492707..1492721 | ^§^TCCCTGAGGCTTCATCTCCTCCTCGGTGTT |  | 47 | 688 |
| △UDC14-ID8 | 1493394..1493380 | *CCGGAATTCTTTGGGAAGGGTCTA | *EcoR*I |  |  |
|  |  |  |  |  |  |
| △UDC15-ID1 | 1666633..1666650 | CCTGATGATTGCGTAAGA |  | 49 | 1983 |
| △UDC15-ID2 | 1668615..1668598 | AGACTGCCTATTGGATTT |  |  |  |
|  |  |  |  |  |  |
| △UDC15-ID3 | 1671387..1671404 | TTACCAAACTGGCTCATC |  | 49 | 2122 |
| △UDC15-ID4 | 1673508 ..1673491 | TAACTCACGGTGTTCTCC |  |  |  |
|  |  |  |  |  |  |
| △UDC15-ID5 | 1667533..1667547 | *TGCACTGCAGGACCGTTAGTTGGAG | *Pst*I | 46 | 808 |
| △UDC15-ID6 | 1668341..1668326 | ^#^CGTTGGGATAAAGTTCTGCCTGACTTGATT |  |  |  |
|  |  |  |  |  |  |
| △UDC15-ID7 | 1672076..1672091 | ^§^AATCAAGTCAGGCAGAACTTTATCCCAACG |  | 46 | 725 |
| △UDC15-ID8 | 1672801..1672787 | *CCGGAATTCTTTGAACTGGCTTTG | *EcoR*I |  |  |
|  |  |  |  |  |  |
| △UDC16-ID1 | 1672088..1672105 | AACGGGCTTGCTCAGAAT |  | 50 | 2238 |
| △UDC16-ID2 | 1674325..1674308 | CGATACCAGCGTAGAGGG |  |  |  |
|  |  |  |  |  |  |
| △UDC16-ID3 | 1676105..1676122 | TTGACATTATTGGAGCCT |  | 50 | 1868 |
| △UDC16-ID4 | 1677972..1677955 | GGTATCGGTGTATTCGTT |  |  |  |
|  |  |  |  |  |  |
| △UDC16-ID5 | 1672775..1672789 | *TGCACTGCAGGGCTTCCTCAATCAA | *Pst*I | 46 | 731 |
| △UDC16-ID6 | 1673506..1673491 | ^#^AGGATTGTGCTCGTACTCACGGTGTTCTCC |  |  |  |
|  |  |  |  |  |  |
| △UDC16-ID7 | 1676398..1676412 | ^§^GGAGAACACCGTGAGTACGAGCACAATCCT |  | 46 | 727 |
| △UDC16-ID8 | 1677124..1677110 | *CCGGAATTCGAGCCACTTTGAAGA | *EcoR*I |  |  |
|  |  |  |  |  |  |
| △UDC20-ID1 | 1925200..1925217 | CGTGGTCCATAACAATCA |  | 52 | 1667 |
| △UDC20-ID2 | 1926866..1926847 | AAGCACAAGCCTTAGTAGAG |  |  |  |
|  |  |  |  |  |  |
| △UDC20-ID3 | 1928066..1928083 | TGTGGCACGGCTTTCTTA |  | 52 | 1768 |
| △UDC20-ID4 | 1929833..1929816 | CATCGGTGGTGACTGGGT |  |  |  |
|  |  |  |  |  |  |
| △UDC20-ID5 | 1926056..1926070 | *TGCACTGCAGCAAACCCTCCTACCC | *Pst*I | 50 | 641 |
| △UDC20-ID6 | 1926696..1926682 | ^#^TGGATGTATTGGCTTGCTCCCTCGCTCTAT |  |  |  |
|  |  |  |  |  |  |
| △UDC20-ID7 | 1928479..1928493 | ^§^ATAGAGCGAGGGAGCAAGCCAATACATCCA |  | 47 | 624 |
| △UDC20-ID8 | 1929102..1929088 | *CGCGGATCCTCTTCCCACAAATCA | *Bam*HI |  |  |

*: restrictive site and the corresponding protective base

#: reverse complementary sequence of ID7

§: reverse complementary sequence of ID6

Supplemental Table 3-1. The information of upregulated DEGs identified in the SC84 2h post-interaction group.

| **Upregulated DEGs identified in the SC84 interaction group** | **Corresponding homologous gene in P1-7 and 89-1591 genomes** | | **Annotation** | **NO. of UDC** | **COG subgroup** |
| --- | --- | --- | --- | --- | --- |
|  | **P1-7** | **89-1591** |  |  |  |
| SSUSC84_0223* | SSU0234 | MUN40_01770 | **Glutamate dehydrogenase^d^** |  | E |
| ^e^SSUSC84_0446c | SSU0462 | MUN40_08150 | BioY family transporter |  | S |
| ^e^SSUSC84_0497* | SSU0512 | MUN40_09290 | Hypothetical protein |  | S |
| ^e^SSUSC84_0674c | SSU0709 | MUN40_04705 | Glycerol-3-phosphate acyltransferase |  | I |
| ^e^SSUSC84_0739b | SSU0776 | MUN40_04365 | Membrane protein |  | S |
| ^e^SSUSC84_0921b | SSU0876 | - | Amino acid ABC transporter substrate-binding protein |  | E |
| ^e^SSUSC84_1405* | SSU1375 | MUN40_07820 | Cystathionine β- lyase | UDC14 | E |
| ^e^SSUSC84_1406a | SSU1376 | MUN40_07825 | Cystathionine γ-synthase |  | E |
| ^e^SSUSC84_1600* | SSU1574 | MUN40_02800 | Methionine ABC transporter permease | UDC15 | E |
| SSUSC84_1601* | SSU1575 | MUN40_02795 | Methionine ABC transporter ATP-binding protein，ATPase component |  | E |
| ^e^SSUSC84_1602* | SSU1576 | MUN40_02790 | Peptidase |  | E |
| SSUSC84_1603c | SSU1577 | MUN40_02785 | MetQ |  | E |
| SSUSC84_1605a | SSU1579 | MUN40_02770 | 5-methyltetrahydropteroyltriglutamate--homocysteine methyltransferase (MetE) | UDC16 | E |
| ^e^SSUSC84_1606a | SSU1580 | MUN40_02765 | 5,10-methylenetetrahydrofolate reductase [NAD(P)H]（MetF） |  | E |
| SSUSC84_1771* | SSU1749 | MUN40_09765 | Dihydroxyacetone kinase subunit DhaK | UDC18 | G |
| SSUSC84_1772* | SSU1750 | MUN40_09770 | Dihydroxyacetone kinase subunit L |  | G |
| SSUSC84_1773* | SSU1751 | MUN40_09775 | PTS-dependent dihydroxyacetone kinase phosphotransferase subunit DhaM |  | G |
| SSUSC84_1774* | SSU1752 | MUN40_09780 | Glycerol transporter |  | G |
| ^e^SSUSC84_1835* | SSU1813 | - | Homocysteine S-methyltransferase（MmuM） | UDC20 | E |
| ^e^SSUSC84_1836* | SSU1814 | - | Amino acid transporter（MmuP） |  | E |
| ^e^SSUSC84_1842c | SSU1820 | MUN40_10160 | Carbonic anhydrase |  | P |

-: absent in the corresponding genome

*: only upregulated in the SC84 interaction group

a: upregulated in three interaction groups

b: upregulated in both SC84 and P1/7 interaction groups

c: upregulated in both SC84 and 89-1591 interaction groups

d: known virulence gene

e: upregulated in the SC84 4h post-interaction group

Supplemental Table 3-2. The information of downregulated DEGs identified in the SC84 2h post-interaction group.

| **Downregulated DEGs identified in the SC84 interaction group** | **Corresponding homologous gene in P1-7 and 89-1591 genomes** | |  |  | **COG subgroup** |
| --- | --- | --- | --- | --- | --- |
|  | **P1-7** | **89-1591** | **Annotation** | **NO. of DDC** |  |
| ^b^SSUSC84_1023**^a^** | SSU0985 | MUN40_06375 | Permease | DDC21 | G |
| ^b^SSUSC84_1024**^a^** | SSU0986 | MUN40_06380 | 2-amino-4-hydroxy-6-hydroxymethyldihydropteridine diphosphokinase |  | H |
| ^b^SSUSC84_1025**^a^** | SSU0987 | MUN40_06385 | Dihydroneopterin aldolase |  | H |
| ^b^SSUSC84_1026**^a^** | SSU0988 | MUN40_06390 | Dihydropteroate synthase |  | H |
| ^b^SSUSC84_1027**^a^** | SSU0989 | MUN40_06395 | GTP cyclohydrolase I FolE |  | H |
| SSUSC84_1422**^a^** | SSU1392 | MUN40_07920 | Pyridoxine ECF transporter | DDC28 | H |
| SSUSC84_1423**^a^** | SSU1393 | MUN40_07925 | Phosphomethylpyrimidine kinase |  | F |

a: downregulated in both SC84 and P1/7 interaction groups

b: downregulated in the SC84 4h post-interaction group

Supplemental Table 4-1. The information of upregulated DEGs identified in the SC84 4h post-interaction group.

| **Upregulated DEGs identified in the SC84 interaction group** | **Corresponding homologous gene in P1-7 and 89-1591 genomes** | | **Annotation** | **NO. of UDC** | **COG subgroup** |
| --- | --- | --- | --- | --- | --- |
|  | **P1-7** | **89-1591** |  |  |  |
| SSUSC84_0026**^a^** | SSU0027 | MUN40_00225 | Phosphoribosylformylglycinamidine synthase | UDC1 | F |
| SSUSC84_0027**^a^** | SSU0028 | MUN40_00230 | Amidophosphoribosyltransferase |  | F |
| SSUSC84_0028**^a^** | SSU0029 | MUN40_00235 | Phosphoribosylformylglycinamidine cyclo-ligase |  | F |
| SSUSC84_0029**^a^** | SSU0030 | MUN40_00240 | Phosphoribosylglycinamide formyltransferase |  | F |
| SSUSC84_0030**^a^** | SSU0031 | MUN40_00245 | Bifunctional phosphoribosylaminoimidazolecarboxamide Formyltransferase/inosine monophosphate cyclohydrolase |  | F |
| SSUSC84_0114* | SSU0119 | MUN40_01145 | HIT family protein | UDC2 | F |
| SSUSC84_0115**^c^** | SSU0120 | MUN40_01150 | Tyrosine--tRNA ligase |  | E |
| SSUSC84_0130**^c^** | SSU0135 | MUN40_01225 | Folate family ECF transporter S component | UDC3 | E |
| SSUSC84_0131**^c^** | SSU0136 | MUN40_01230 | Folylpolyglutamate synthase |  | E |
| SSUSC84_0133**^c^** | SSU0139 | MUN40_01240 | Hypothetical protein | UDC4 | S |
| SSUSC84_0134**^c^** | SSU0140 | MUN40_01245 | Thiol reductase thioredoxin |  | O |
| SSUSC84_0135**^c^** | SSU0141 | MUN40_01250 | Hypothetical protein |  | S |
| SSUSC84_0136**^c^** | SSU0142 | MUN40_01255 | tRNA-binding protein |  | J |
| SSUSC84_0212* | SSU0223 | MUN40_01680 | Thiol reductase thioredoxin |  | O |
| SSUSC84_0219* | SSU0230 | MUN40_01750 | Sodium:alanine symporter |  | E |
| SSUSC84_0265**^a^** | SSU0276 | MUN40_01980 | **38 kDa^d^** |  | S |
| SSUSC84_0350**^a^** | SSU0364 | MUN40_00450 | Sulfurtransferase |  | S |
| SSUSC84_0354**^c^** | SSU0368 | MUN40_00470 | Cold-shock protein |  | K |
| SSUSC84_0408**^a^** | SSU0423 | MUN40_00790 | Hypothetical protein | UDC5 | S |
| SSUSC84_0409**^a^** | SSU0424 | MUN40_00795 | S26 family signal peptidase |  | U |
| SSUSC84_0427**^b^** | SSU0443 | MUN40_08240 | NUDIX hydrolase |  | L |
| SSUSC84_0431c | SSU0447 | MUN40_08220 | Peptide ABC transporter ATP-binding protein |  | E |
| SSUSC84_0444* | SSU0460 | MUN40_08160 | Hypothetical protein | UDC6 | S |
| SSUSC84_0445* | SSU0461 | MUN40_08155 | Hypothetical protein |  | S |
| SSUSC84_0446**^a^** | SSU0462 | MUN40_08150 | BioY family transporter |  | S |
| SSUSC84_0453* | SSU0469 | - | Hypothetical protein |  | S |
| SSUSC84_0491**^c^** | SSU0506 | MUN40_09320 | Hydrolase |  | S |
| SSUSC84_0497**^a^** | SSU0512 | MUN40_09290 | Hypothetical protein |  | S |
| SSUSC84_0564* | SSU0591 | MUN40_05210 | Glucosamine-6-phosphate deaminase |  | G |
| SSUSC84_0594* | SSU0621 | MUN40_05035 | Phosphoserine aminotransferase | UDC7 | E |
| SSUSC84_0595* | SSU0622 | MUN40_05030 | GNAT family acetyltransferase |  | E |
| SSUSC84_0596* | SSU0623 | MUN40_05025 | 3-phosphoglycerate dehydrogenase |  | E |
| SSUSC84_0597* | SSU0624 | MUN40_05020 | Methylated-DNA--protein-cysteine methyltransferase |  | E |
| SSUSC84_0598* | SSU0625 | MUN40_05015 | Arsenate reductase family protein |  | E |
| SSUSC84_0612**^a^** | SSU0645 | MUN40_04935 | Pantothenic acid transporter pant | UDC8 | I |
| SSUSC84_0613**^a^** | SSU0646 | MUN40_04930 | Phosphopantothenoylcysteine decarboxylase |  | E |
| SSUSC84_0614**^a^** | SSU0647 | MUN40_04925 | Phosphopantothenate--cysteine ligase |  | E |
| SSUSC84_0617**^b^** | SSU0650 | MUN40_04910 | Pyridine nucleotide-disulfide oxidoreductase |  | C |
| SSUSC84_0648**^a^** | SSU0682 | MUN40_04795 | NADH oxidase |  | S |
| SSUSC84_0662**^c^** | SSU0696 | MUN40_04770 | Holin |  | S |
| SSUSC84_0674**^a^** | SSU0709 | MUN40_04705 | Glycerol-3-phosphate acyltransferase |  | I |
| SSUSC84_0723* | SSU0759 | MUN40_04445 | Endonuclease III |  | L |
| SSUSC84_0739**^b^** | SSU0776 | MUN40_04365 | Hypothetical protein | UDC9 | S |
| SSUSC84_0740**^b^** | SSU0777 | MUN40_04360 | Hypothetical protein |  | S |
| SSUSC84_0763* | SSU0800 | MUN40_04250 | Hypothetical protein |  | S |
| SSUSC84_0893**^c^** | SSU0847 | MUN40_05595 | DNA topoisomerase I |  | L |
| SSUSC84_0899* | SSU0853 | MUN40_05625 | Hypothetical protein | UDC10 | K |
| SSUSC84_0900**^b^** | SSU0854 | MUN40_05630 | Hemolysin III |  | S |
| SSUSC84_0920**^c^** | SSU0875 | MUN40_05730 | Amino acid ABC transporter substrate-binding protein | UDC11 | E |
| SSUSC84_0921**^b^** | SSU0876 | - | Amino acid ABC transporter substrate-binding protein |  | E |
| SSUSC84_0924**^a^** | SSU0879 | MUN40_05760 | **IgA1^d^** |  | E |
| SSUSC84_0928**^c^** | SSU0883 | MUN40_05775 | Amino acid ABC transporter permease |  | E |
| SSUSC84_1018**^c^** | SSU0980 | MUN40_06350 | Membrane protein | UDC12 | S |
| SSUSC84_1019**^c^** | SSU0981 | MUN40_06355 | Transcriptional regulator |  | K |
| SSUSC84_1058**^a^** | SSU1020 | MUN40_06550 | F0F1 ATP synthase subunit C |  | C |
| SSUSC84_1081* | SSU1043 | MUN40_06665 | NrdH-redoxin |  | O |
| SSUSC84_1167**^a^** | SSU1134 | MUN40_03450 | ZIP family metal transporter |  | P |
| SSUSC84_1246**^a^** | SSU1213 | MUN40_03040 | 2-Cys peroxiredoxin |  | O |
| SSUSC84_1286**^c^** | SSU1257 | MUN40_07040 | Large-conductance mechanosensitive channel |  | M |
| SSUSC84_1338**^c^** | SSU1308 | MUN40_07520 | 6-phospho-beta-glucosidase | UDC13 | G |
| SSUSC84_1339**^b^** | SSU1309 | MUN40_07305 | PTS beta-glucoside transporter subunit EIIBCA |  | G |
| SSUSC84_1340**^b^** | SSU1310 | MUN40_07310 | Transcription antiterminator BglG |  | K |
| SSUSC84_1346**^a^** | SSU1316 | MUN40_07340 | Hypothetical protein |  | S |
| SSUSC84_1386**^b^** | SSU1356 | MUN40_07725 | Superoxide dismutase |  | P |
| SSUSC84_1394**^c^** | SSU1364 | MUN40_07765 | Branched-chain amino acid ABC transporter substrate-binding protein |  | E |
| SSUSC84_1405**^b^** | SSU1375 | MUN40_07820 | Cystathionine β- lyase | UDC14 | E |
| SSUSC84_1406**^a^** | SSU1376 | MUN40_07825 | Cystathionine γ-synthase |  | E |
| SSUSC84_1545**^a^** | SSU1519 | MUN40_08550 | Diaminopimelate decarboxylase |  | E |
| SSUSC84_1549* | SSU1523 | MUN40_08570 | 23S rRNA methyltransferase |  | J |
| SSUSC84_1565* | SSU1539 | MUN40_08655 | AI-2E family transporter |  | S |
| SSUSC84_1577**^a^** | SSU1551 | MUN40_02905 | Transcriptional regulator |  | K |
| SSUSC84_1594* | SSU1568 | MUN40_02830 | Hypothetical protein |  | S |
| SSUSC84_1600**^b^** | SSU1574 | MUN40_02800 | Methionine ABC transporter permease |  | E |
| SSUSC84_1602**^c^** | SSU1576 | MUN40_02790 | Peptidase |  | E |
| SSUSC84_1606**^a^** | SSU1580 | MUN40_02765 | 5,10-methylenetetrahydrofolate reductase （MetF） |  | E |
| SSUSC84_1653**^c^** | SSU1628 | MUN40_02510 | 30S ribosomal protein S18 |  | J |
| SSUSC84_1655**^c^** | SSU1630 | MUN40_02500 | 30S ribosomal protein S6 |  | J |
| SSUSC84_1681* | SSU1656 | MUN40_02280 | Dihydroxyacetone kinase | UDC17 | C |
| SSUSC84_1682**^a^** | SSU1657 | MUN40_02275 | Hypothetical protein |  | S |
| SSUSC84_1750* | SSU1728 | MUN40_09640 | 30S ribosomal protein S15 |  | J |
| SSUSC84_1785**^b^** | SSU1763 | - | **DNA mismatch repair protein MutT^d^** | UDC19 | F |
| SSUSC84_1786**^b^** | SSU1764 | - | **Nicotinamide mononucleotide transporter^d^** |  | H |
| SSUSC84_1787**^b^** | SSU1765 | - | **Transcriptional regulator^d^** |  | H |
| SSUSC84_1799**^c^** | SSU1777 | MUN40_09920 | Penicillin-binding protein 2A |  | M |
| SSUSC84_1832* | SSU1810 | MUN40_10120 | DNA-binding protein |  | S |
| SSUSC84_1835**^b^** | SSU1813 | - | Homocysteine S-methyltransferase | UDC20 | E |
| SSUSC84_1836**^b^** | SSU1814 | - | Amino acid transporter |  | E |
| SSUSC84_1841**^a^** | SSU1819 | MUN40_10155 | TIGR00266 family protein | UDC21 | S |
| SSUSC84_1842**^a^** | SSU1820 | MUN40_10160 | Carbonic anhydrase |  | P |
| SSUSC84_1851* | SSU1829 | MUN40_10205 | Rhomboid family intramembrane serine protease | UDC22 | E |
| SSUSC84_1852* | SSU1830 | MUN40_10210 | 5-formyltetrahydrofolate cyclo-ligase |  | E |
| SSUSC84_1854* | SSU1832 | MUN40_10220 | 2,3,4,5-tetrahydropyridine-2,6-dicarboxylate N-acetyltransferase |  | E |
| SSUSC84_1857**^c^** | SSU1835 | MUN40_10230 | Phosphoesterase |  | T |
| SSUSC84_1861**^a^** | SSU1839 | MUN40_10255 | Transketolase |  | G |
| SSUSC84_1923**^c^** | SSU1905 | MUN40_10845 | Hypothetical protein |  | S |
| SSUSC84_1950* | SSU1932 | MUN40_10945 | Phosphoesterase | UDC23 | S |
| SSUSC84_1951* | SSU1933 | MUN40_10950 | Flavocytochrome c |  | C |
| SSUSC84_1953**^c^** | SSU1935 | MUN40_11045 | 30S ribosomal protein S4 |  | J |
| SSUSC84_1981* | SSU1963 | MUN40_11260 | ABC-F family ATPase |  | S |

-: absent in the corresponding genome

*: only upregulated in the SC84 interaction group

a: upregulated in three interaction groups

b: upregulated in both SC84 and P1/7 interaction groups

c: upregulated in both SC84 and 89-1591 interaction groups

d: known virulence gene

Supplemental Table 4-2. The information of downregulated DEGs identified in the SC84 4h post-interaction group.

| **Downregulated DEGs identified in the SC84 interaction group** | **Corresponding homologous gene in P1-7 and 89-1591 genomes** | | **Annotation** | **NO. of DDC** | **COG subgroup** |
| --- | --- | --- | --- | --- | --- |
|  | **P1-7** | **89-1591** |  |  |  |
| SSUSC84_0064c | SSU0068 | MUN40_00860 | Putative competence-specific global transcription modulator (fragment) |  | K |
| SSUSC84_0104b | SSU0109 | - | Hypothetical protein |  | S |
| SSUSC84_0112b | SSU0117 | - | Hypothetical protein |  | S |
| SSUSC84_0177c | SSU0185 | MUN40_01460 | Tagatose-6-phosphate ketose | DDC1 | G |
| SSUSC84_0178* | SSU0186 | - | **Hp197^d^** |  | S |
| SSUSC84_0186* | SSU0195 | MUN40_01505 | Tagatose-6-phosphate kinase |  | G |
| SSUSC84_0189c | SSU0198 | MUN40_01520 | Repressor |  | G |
| SSUSC84_0190a | SSU0199 | MUN40_01525 | PTS cellobiose transporter subunit IIC | DDC2 | G |
| SSUSC84_0191c | SSU0200 | MUN40_01530 | DUF4838 domain-containing protein |  | S |
| SSUSC84_0192c | SSU0201 | MUN40_01535 | Sugar ABC transporter substrate-binding protein |  | G |
| SSUSC84_0193* | SSU0202 | MUN40_01540 | Hypothetical protein |  | S |
| SSUSC84_0194c | SSU0203 | MUN40_01545 | Nitrate ABC transporter permease |  | P |
| SSUSC84_0195* | SSU0204 | MUN40_01550 | ATP-binding protein |  | P |
| SSUSC84_0268c | SSU0279 | MUN40_01995 | **Nucleotide exchange factor GrpE^d^** | DDC3 | O |
| SSUSC84_0269^c^ | SSU0280 | MUN40_02000 | Molecular chaperone DnaK |  | O |
| SSUSC84_0314* | SSU0327 | - | Deoxyguanosinetriphosphate triphosphohydrolase | DDC4 | F |
| SSUSC84_0315* | SSU0328 | MUN40_09355 | LacI family transcriptional regulator |  | K |
| SSUSC84_0316c | SSU0329 | MUN40_09350 | Galactokinase |  | G |
| SSUSC84_0317a | SSU0330 | MUN40_09345 | Galactose-1-phosphate uridylyltransferase |  | G |
| SSUSC84_0320c | SSU0333 | MUN40_03650 | Hypothetical protein |  | S |
| SSUSC84_0328a | SSU0342 | MUN40_03690 | Ribosome silencing factor RsfS |  | J |
| SSUSC84_0330* | SSU0344 | - | Hypothetical protein |  | K |
| SSUSC84_0332c | SSU0346 | MUN40_03715 | MerR family transcriptional regulator |  | K |
| SSUSC84_0339a | SSU0353 | MUN40_00395 | 4-alpha-glucanotransferase | DDC5 | G |
| SSUSC84_0340a | SSU0354 | MUN40_00400 | Maltodextrin phosphorylase |  | G |
| SSUSC84_0349* | SSU0363 | MUN40_00445 | Hypothetical protein |  | S |
| SSUSC84_0379c | SSU0394 | MUN40_00580 | Amidophosphoribosyltransferase | DDC6 | F |
| SSUSC84_0380c | SSU0395 | MUN40_00585 | Ribosomal subunit interface protein |  | J |
| SSUSC84_0387a | SSU0402 | MUN40_00690 | Beta-galactosidase | DDC7 | G |
| SSUSC84_0388a | SSU0403 | MUN40_00695 | PTS mannose transporter subunit IID |  | G |
| SSUSC84_0389a | SSU0404 | MUN40_00700 | PTS fructose transporter subunit IIC |  | G |
| SSUSC84_0390a | SSU0405 | MUN40_00705 | PTS fructose transporter subunit IID |  | G |
| SSUSC84_0391a | SSU0406 | MUN40_00710 | PTS fructose transporter subunit IIA |  | G |
| SSUSC84_0392a | SSU0407 | MUN40_00715 | Galactose mutarotase |  | G |
| SSUSC84_0398* | SSU0413 | - | Hypothetical protein |  | S |
| SSUSC84_0464a | SSU0480 | MUN40_08045 | DNA-directed RNA polymerase sigma-70 factor | DDC8 | K |
| SSUSC84_0465a | SSU0481 | MUN40_08040 | Hypothetical protein |  | S |
| SSUSC84_0507c | SSU0523 | MUN40_09240 | **Glycosyl transferase^e^** | DDC9 | M |
| SSUSC84_0508c | SSU0524 | MUN40_09235 | **Polymerase WZY^e^** |  | M |
| SSUSC84_0509* | SSU0525 | MUN40_09230 | **Glycosyl transferase^e^** |  | M |
| SSUSC84_0532c | SSU0556 | MUN40_03725 | **Hypothetical protein^e^** |  | M |
| SSUSC84_0547a | SSU0571 | MUN40_05300 | Hypothetical protein | DDC10 | S |
| SSUSC84_0548a | SSU0572 | MUN40_05295 | Hypothetical protein |  | V |
| SSUSC84_0555c | SSU0580 | MUN40_05260 | **Arginine deiminase^d^** | DDC11 | E |
| SSUSC84_0556c | SSU0581 | MUN40_05255 | N-acetyltransferase |  | E |
| SSUSC84_0557a | SSU0582 | MUN40_05250 | Ornithine carbamoyltransferase |  | E |
| SSUSC84_0558a | SSU0583 | MUN40_05245 | Carbamate kinase |  | E |
| SSUSC84_0559a | SSU0584 | MUN40_05240 | Arginine:ornithine antiporter |  | E |
| SSUSC84_0560a | SSU0585 | MUN40_05235 | Hypothetical protein |  | E |
| SSUSC84_0619* | SSU0652 | - | Hypothetical protein | DDC12 | V |
| SSUSC84_0620* | SSU0653 | - | Type I restriction modification protein |  | V |
| SSUSC84_0621* | SSU0654 | - | Hypothetical protein |  | S |
| SSUSC84_0622* | SSU0655 | - | Hypothetical protein |  | S |
| SSUSC84_0636c | SSU0670 | MUN40_04830 | Glycerol dehydrogenase |  | C |
| SSUSC84_0661* | SSU0695 | MUN40_04775 | Abortive infection protein |  | S |
| SSUSC84_0687* | SSU0723 | MUN40_04635 | Hypothetical protein | DDC13 | S |
| SSUSC84_0688c | SSU0724 | MUN40_04630 | Hypothetical protein |  | S |
| SSUSC84_0765c | SSU0802 | MUN40_04240 | ATPase AAA | DDC14 | L |
| SSUSC84_0766c | SSU0803 | MUN40_04235 | 5-methylcytosine-specific restriction system specificity protein McrC |  | V |
| SSUSC84_0768* | SSU0805 | - | Hypothetical protein |  | S |
| SSUSC84_0772* | SSU0809 | - | Hypothetical protein |  | S |
| SSUSC84_0807c | - | MUN40_04100 | **Site-specific integrase^f^** |  | L |
| SSUSC84_0819* | - | - | **Chromosome segregation ATPase^f^** |  | U |
| SSUSC84_0857* | - | - | **Hypothetical protein^f^** | DDC15 | L |
| SSUSC84_0858* | - | - | **Recombinase^f^** |  | L |
| SSUSC84_0859* | - | - | **Serine recombinase^f^** |  | L |
| SSUSC84_0861* | - | - | **Hypothetical protein^f^** | DDC16 | L |
| SSUSC84_0862* | - | - | **Methyltransferase^f^** |  | Q |
| SSUSC84_0863* | - | - | **Aminoglycoside nucleotidyltransferase ANT(6)-Ia^f^** |  | S |
| SSUSC84_0864* | - | - | **Adenine phosphoribosyltransferase^f^** |  | F |
| SSUSC84_0868* | - | - | **Hypothetical protein^f^** |  | S |
| SSUSC84_0870* | - | MUN40_03895 | **Hypothetical protein^f^** |  | L |
| SSUSC84_0874c | - | MUN40_03830 | **Transcriptional regulator^f^** | DDC17 | K |
| SSUSC84_0875c | - | MUN40_03825 | **Abortive infection protein^f^** |  | S |
| SSUSC84_0939c | SSU0894 | MUN40_05955 | Transcriptional antiterminator | DDC18 | K |
| SSUSC84_0940b | SSU0895 | MUN40_05960 | Tagatose 1,6-diphosphate aldolase |  | G |
| SSUSC84_0941* | SSU0894 | MUN40_05965 | Tagatose-6-phosphate kinase lacC |  | G |
| SSUSC84_0942* | SSU0898 | MUN40_05970 | Galactose-6-phosphate isomerase subunit LacB |  | G |
| SSUSC84_0943* | SSU0899 | MUN40_05975 | Galactose-6-phosphate isomerase subunit LacA |  | G |
| SSUSC84_0945c | SSU0901 | MUN40_05980 | DeoR family transcriptional regulator |  | K |
| SSUSC84_0980c | SSU0939 | MUN40_06165 | Type I pantothenate kinase |  | F |
| SSUSC84_0983* | SSU0943 | MUN40_06175 | Transglutaminase | DDC19 | D |
| SSUSC84_0984c | SSU0944 | MUN40_06180 | **Two-component sensor histidine kinase^d^** |  | T |
| SSUSC84_0985* | SSU0945 | MUN40_06185 | **DNA-binding response regulator^d^** |  | T |
| SSUSC84_0986* | SSU0946 | MUN40_06190 | Hypothetical protein |  | S |
| SSUSC84_1007* | SSU0969 | MUN40_06285 | Hypothetical protein | DDC20 | S |
| SSUSC84_1008* | SSU0970 | MUN40_06290 | Hypothetical protein |  | S |
| SSUSC84_1009* | SSU0971 | MUN40_06295 | ATPase |  | S |
| SSUSC84_1010* | SSU0972 | MUN40_06300 | Haloacid dehalogenase |  | S |
| SSUSC84_1023b | SSU0985 | MUN40_06375 | Cytochrome C biogenesis protein CcmE | DDC21 | G |
| SSUSC84_1024b | SSU0986 | MUN40_06380 | 2-amino-4-hydroxy-6-hydroxymethyldihydropteridine diphosphokinase |  | H |
| SSUSC84_1025b | SSU0987 | MUN40_06385 | Dihydroneopterin aldolase |  | H |
| SSUSC84_1026b | SSU0988 | MUN40_06390 | Dihydropteroate synthase |  | H |
| SSUSC84_1027b | SSU0989 | MUN40_06395 | GTP cyclohydrolase I FolE |  | H |
| SSUSC84_1029c | SSU0991 | MUN40_06405 | DUF4956 domain-containing protein | DDC22 | S |
| SSUSC84_1030c | SSU0992 | MUN40_06410 | Transporter |  | P |
| SSUSC84_1036a | SSU0998 | MUN40_06440 | Glycosyl hydrolase | DDC23 | G |
| SSUSC84_1037a | SSU0999 | MUN40_06445 | Beta-hexosamidase |  | G |
| SSUSC84_1038a | SSU1000 | MUN40_06450 | Phosphatase |  | G |
| SSUSC84_1039a | SSU1001 | MUN40_06455 | Dioxygenase |  | G |
| SSUSC84_1040a | SSU1002 | MUN40_06460 | Mannonate dehydratase |  | G |
| SSUSC84_1041c | SSU1003 | MUN40_06465 | Uronate isomerase |  | G |
| SSUSC84_1087b | SSU1050 | MUN40_06700 | Hyaluronidase | DDC24 | N |
| SSUSC84_1088c | SSU1054 | MUN40_06710 | Preprotein translocase subunit YajC |  | U |
| SSUSC84_1089c | SSU1055 | MUN40_06715 | PTS N-acetylglucosamine transporter subunit IIBC |  | G |
| SSUSC84_1090c | SSU1056 | MUN40_06720 | PTS N-acetylgalactosamine transporter subunit IIC |  | G |
| SSUSC84_1091c | SSU1057 | MUN40_06725 | PTS N-acetylgalactosamine transporter subunit IIB |  | G |
| SSUSC84_1092c | SSU1058 | MUN40_06730 | Glucuronyl hydrolase |  | G |
| SSUSC84_1093a | SSU1059 | MUN40_06735 | PTS N-acetylgalactosamine transporter subunit IIA |  | G |
| SSUSC84_1102c | SSU1069 | MUN40_06780 | Hypothetical protein |  | S |
| SSUSC84_1158c | SSU1125 | MUN40_03495 | Sulfatase |  | M |
| SSUSC84_1176c | SSU1143 | MUN40_03405 | **Zinc carboxypeptidase^d^** |  | E |
| SSUSC84_1247a | SSU1214 | MUN40_03035 | Copper-translocating P-type ATPase | DDC25 | P |
| SSUSC84_1248c | SSU1215 | MUN40_03030 | Peptidase C69 |  | E |
| SSUSC84_1269c | SSU1237 | MUN40_07945 | **Ferrous iron transport protein B^d^** |  | P |
| SSUSC84_1333* | SSU1303 | MUN40_07265 | Lipoprotein |  | S |
| SSUSC84_1375* | SSU1345 | - | Transcriptional regulator | DDC26 | K |
| SSUSC84_1376* | SSU1346 | - | Hypothetical protein |  | S |
| SSUSC84_1399a | SSU1369 | MUN40_07790 | **Sucrose phosphorylase^d^** | DDC27 | G |
| SSUSC84_1400a | SSU1370 | MUN40_07795 | Sugar ABC transporter permease |  | G |
| SSUSC84_1401a | SSU1371 | MUN40_07800 | Sugar ABC transporter permease |  | G |
| SSUSC84_1402a | SSU1372 | MUN40_07805 | Sugar ABC transporter substrate-binding protein |  | G |
| SSUSC84_1403a | SSU1373 | MUN40_07810 | Alpha-galactosidase |  | G |
| SSUSC84_1664* | SSU1639 | MUN40_02365 | Lipoprotein |  | S |
| SSUSC84_1724* | SSU1701 | MUN40_09600 | Sugar ABC transporter ATP-binding protein |  | G |
| SSUSC84_1746* | SSU1724 | - | Membrane protein |  | S |
| SSUSC84_1859* | SSU1837 | MUN40_10240 | Hypothetical protein |  | S |
| SSUSC84_1901* | SSU1879 | MUN40_10430 | **2',3'-cyclic-nucleotide 2'-phosphodiesterase^d^** |  | F |
| SSUSC84_1933c | SSU1915 | MUN40_10885 | Sugar ABC transporter substrate-binding protein | DDC29 | G |
| SSUSC84_1934c | SSU1916 | MUN40_10890 | Sugar ABC transporter permease |  | G |
| SSUSC84_1985c | SSU1968 | MUN40_11295 | Serine protease | DDC30 | O |
| SSUSC84_1986c | SSU1969 | MUN40_11300 | Chromosome partitioning protein |  | K |

-: absent in the corresponding genome

*: only downregulated in the SC84 interaction group

a: downregulated in three interaction groups

b: downregulated in both SC84 and P1/7 interaction groups

c: downregulated in both SC84 and 89-1591 interaction groups

d: known virulence gene

e: gene of *cps* locus

f: gene of 89K PAI

Supplemental Table 5. The regulation results of known virulence genes in the SC84 interaction groups.

| **Known virulence genes** | **Gene number in SC84 genome** | **Gene number in P1-7 genome** | **Gene number in 89-1591 genome** | **2h post-interaction group** | **4h post-interaction group** |
| --- | --- | --- | --- | --- | --- |
| *purD* | SSUSC84_0031 | SSU0032 | MUN40_00270 | / | / |
| *05SSU0053* | SSUSC84_0046 | SSU0049 | MUN40_00355 | / | / |
| *adcR* | SSUSC84_0107 | SSU0112 | MUN40_01115 | / | / |
| *impdh2* | SSUSC84_135 | SSU0141 | MUN40_01250 | / | / |
| *EF-G* | SSUSC84_144 | SSU0151 | MUN40_01295 | / | / |
| *sspep* | SSUSC84_145 | SSU0152 | MUN40_01300 | / | /& |
| *pgk* | SSUSC84_147 | SSU0154 | MUN40_01310 | / | / |
| *glnA* | SSUSC84_0150 | SSU0157 | MUN40_01325 | / | / |
| *ef* | SSUSC84_0164 | SSU0171 | - | /& | / |
| *hp197* | SSUSC84_0178 | SSU0186 | - | /& | ↓ |
| *dppIV* | SSUSC84_0179 | SSU0187 | MUN40_01470 | / | / |
| *copper-exporting ATPase* | SSUSC84_0198 | SSU0207 | MUN40_01565 | / | / |
| *treR* | SSUSC84_0213 | SSU0224 | MUN40_01690 | / | / |
| *hp0245* | SSUSC84_0216 | SSU0227 | MUN40_01735 | / | / |
| *glutamate dehydrogenase* | SSUSC84_0223 | SSU0234 | MUN40_01770 | ↑ | / |
| *hp272* | SSUSC84_0242 | SSU0253 | MUN40_01860 | /& | /& |
| *adhE* | SSUSC84_250 | SSU0261 | MUN40_01900 | / | / |
| *38 kDa-1* | SSUSC84_0265 | SSU0275 | MUN40_01980 | / | ↑ |
| *38 kDa-2* | SSUSC84_0266 | SSU0277 | MUN40_01985 | / | / |
| *hrcA* | SSUSC84_267 | SSU0278 | MUN40_01990 | / | / |
| *grpE* | SSUSC84_268 | SSU0279 | MUN40_01995 | / | ↓ |
| *perR* | SSUSC84_0278 | SSU0289 | MUN40_02055 | / | / |
| *Tig* | SSUSC84_0294 | SSU0306 | MUN40_02125 | / | / |
| *103/adhesion protein* | SSUSC84_0296 | SSU0308 | MUN40_09505 | / | / |
| *htpS* | SSUSC84_0297 | SSU0309 | MUN40_09500 | / | / |
| *fba* | SSUSC84_300 | SSU0312 | MUN40_09470 | / | / |
| *codY* | SSUSC84_307 | SSU0320 | MUN40_09400 | / | / |
| *luxS* | SSUSC84_0362 | SSU0376 | MUN40_00515 | / | / |
| *stp* | SSUSC84_0369 | SSU0383 | MUN40_00550 | / | / |
| *stk* | SSUSC84_370 | SSU0384 | MUN40_00555 | / | / |
| *vrasr* | SSUSC84_373 | SSU0388 | MUN40_10825 | / | / |
| *BgaC* | SSUSC84_387 | SSU0402 | MUN40_00690 | / | / |
| *tran* | SSUSC84_403 | SSU0418 | - | / | / |
| *SSU05_0473* | SSUSC84_0410 | SSU0425 | MUN40_00800 | / | / |
| *srtF* | SSUSC84_0412 | SSU0428 | MUN40_08320 | / | / |
| *collagenase* | SSUSC84_0441 | SSU0457 | MUN40_08170 | / | / |
| *peptidase* | SSUSC84_0442 | SSU0458 | MUN40_08165 | / | / |
| *EF-Tu* | SSUSC84_466 | SSU0482 | MUN40_08035 | / | / |
| *pk* | SSUSC84_479 | SSU0495 | MUN40_06960 | / | / |
| *ides* | SSUSC84_480 | SSU0496 | MUN40_06955 | / | / |
| *permease* | SSUSC84_0485 | SSU0501 | MUN40_06920 | / | / |
| *gntR* | SSUSC84_498 | SSU0513 | MUN40_09285 | / | / |
| *neuB* | SSUSC84_0517 | SSU0535 | MUN40_09195 | / | / |
| *neuC* | SSUSC84_0518 | SSU0536 | MUN40_09190 | / | / |
| *Flps* | SSUSC84_554 | SSU0579 | MUN40_05265 | / | / |
| *arcA* | SSUSC84_0555 | SSU0580 | MUN40_05260 | / | ↓ |
| *arcH* | SSUSC84_0561 | SSU0587 | MUN40_05225 | / | / |
| *argR* | SSUSC84_0562 | SSU0588 | MUN40_05220 | / | / |
| *dltA* | SSUSC84_0569 | SSU0596 | MUN40_05160 | / | / |
| *05SSU0660* | SSUSC84_0589 | SSU0616 | MUN40_05060 | / | / |
| *mrp* | SSUSC84_0671 | SSU0706 | MUN40_04720 | / | / |
| *sspA* | SSUSC84_0721 | SSU0757 | MUN40_04455 | / | / |
| *clpX* | SSUSC84_744 | SSU0781 | MUN40_04345 | / | / |
| *guaA* | SSUSC84_0779 | SSU0816 | MUN40_04230 | / | / |
| *SpyM3-0908* | SSUSC84_0797 | SSU0835 | MUN40_04075 | / | / |
| *nisK* | SSUSC84_0810 | - | - | / | / |
| *nisR* | SSUSC84_0811 | - | - | / | / |
| *hhly3* | SSUSC84_830 | - | - | / | / |
| *sp1* | SSUSC84_847 | - | - | / | / |
| *salR* | SSUSC84_0849 | - | - | / | / |
| *salK* | SSUSC84_0850 | - | - | / | / |
| *traG* | SSUSC84_0881 | - | MUN40_05540 | / | / |
| *Ssads* | SSUSC84_0906 | SSU0860 | MUN40_05660 | / | / |
| *igA1* | SSUSC84_0924 | SSU0879 | MUN40_05760 | / | ↑ |
| *relQ* | SSUSC84_0959 | SSU0916 | MUN40_06050 | / | / |
| *rex* | SSUSC84_0965 | SSU0922 | MUN40_06080 | / | / |
| *srtA* | SSUSC84_0968 | SSU0925 | MUN40_06085 | / | / |
| *hp1083* | SSUSC84_0975 | SSU0934 | MUN40_06140 | / | / |
| *cdd* | SSUSC84_0976 | SSU0935 | MUN40_06145 | / | / |
| *cia*H | SSUSC84_0984 | SSU0944 | MUN40_06180 | / | ↓ |
| *cia*R | SSUSC84_0985 | SSU0945 | MUN40_06185 | /& | ↓ |
| *pept* | SSUSC84_1011 | SSU0973 | MUN40_06305 | / | / |
| *prsa* | SSUSC84_1111 | SSU1078 | MUN40_06820 | / | / |
| *pepf* | SSUSC84_1115 | SSU1082 | MUN40_06840 | / | / |
| *autolysin* | SSUSC84_1160 | SSU1127 | MUN40_03485 | / | / |
| *SSU05_1311* | SSUSC84_1176 | SSU1143 | MUN40_03405 | / | ↓ |
| *covR* | SSUSC84_1224 | SSU1191 | MUN40_03155 | / | / |
| *sao* | SSUSC84_1234 | SSU1201 | MUN40_03105 | / | / |
| *ccpA* | SSUSC84_1235 | SSU1202 | MUN40_03100 | / | / |
| *sly* | SSUSC84_1264 | SSU1231 | - | / | / |
| *feoB* | SSUSC84_1269 | SSU1237 | MUN40_07945 | / | ↓ |
| *fbp* | SSUSC84_1341 | SSU1311 | MUN40_07315 | / | / |
| *eno* | SSUSC84_1350 | SSU1320 | MUN40_07350 | / | / |
| *hp1538* | SSUSC84_1385 | SSU1355 | MUN40_07720 | / | / |
| *clpP* | SSUSC84_1396 | SSU1366 | MUN40_07775 | / | / |
| *gtfA* | SSUSC84_1399 | SSU1369 | MUN40_07790 | / | ↓ |
| *lgt* | SSUSC84_1448 | SSU1418 | MUN40_08825 | / | / |
| *pgdA* | SSUSC84_1477 | SSU1448 | MUN40_08985 | / | / |
| *pgm* | SSUSC84_1480 | SSU1451 | MUN40_08995 | / | / |
| *ihk* | SSUSC84_1499 | SSU1471 | MUN40_09100 | / | / |
| *irr* | SSUSC84_1500 | SSU1472 | MUN40_09105 | / | / |
| *ofs* | SSUSC84_1502 | SSU1474 | - | / | / |
| *yzpa* | SSUSC84_1503 | SSU1476 | MUN40_08335 | / | / |
| *dpr* | SSUSC84_1513 | SSU1487 | MUN40_08385 | / | / |
| *ham1* | SSUSC84_1542 | SSU1516 | MUN40_08535 | / | / |
| *hp1717* | SSUSC84_1553 | SSU1527 | MUN40_08590 | / | / |
| *mannose-specific EIIAB: Phosphotransferase* | SSUSC84_1609 | SSU1583 | MUN40_02750 | / | / |
| *manM* | SSUSC84_1610 | SSU1584 | MUN40_02745 | / | / |
| *manN* | SSUSC84_1611 | SSU1585 | MUN40_02740 | / | / |
| *manO* | SSUSC84_1612 | SSU1586 | MUN40_02735 | / | / |
| *SerS* | SSUSC84_1613 | SSU1587 | MUN40_02730 | / | / |
| *virA* | SSUSC84_1615 | SSU1589 | - | / | / |
| *kar* | SSUSC84_1628 | SSU1603 | MUN40_02630 | / | / |
| *IgdE* | SSUSC84_1641 | SSU1616 | MUN40_02570 | / | / |
| *scrB* | SSUSC84_1644 | SSU1619 | MUN40_02555 | / | / |
| *scrA* | SSUSC84_1645 | SSU1620 | MUN40_02550 | / | / |
| *oppa* | SSUSC84_1688 | SSU1664 | MUN40_02245 | / | / |
| *1910HR* | SSUSC84_1726 | SSU1703 | - | / | / |
| *1910HK* | SSUSC84_1727 | SSU1704 | - | / | / |
| *gp92* | SSUSC84_1737 | SSU1714 | - | / | / |
| *endo D* | SSUSC84_1738 | SSU1715 | - | / | / |
| *purA* | SSUSC84_1780 | SSU1758 | MUN40_09810 | / | / |
| *SsnA* | SSUSC84_1782 | SSU1760 | - | / | / |
| *mutT* | SSUSC84_1785 | SSU1763 | - | / | ↑ |
| *pnuc* | SSUSC84_1786 | SSU1764 | - | / | ↑ |
| *nadR* | SSUSC84_1787 | SSU1765 | - | / | ↑ |
| *rgg* | SSUSC84_1811 | SSU1789 | MUN40_10030 | / | / |
| *apuA* | SSUSC84_1871 | SSU1849 | MUN40_10305 | / | / |
| *troA* | SSUSC84_1891 | SSU1869 | MUN40_10405 | / | / |
| *revS* | SSUSC84_1894 | SSU1872 | - | / | / |
| *relA* | SSUSC84_1900 | SSU1878 | MUN40_10425 | / | / |
| *sntA* | SSUSC84_1901 | SSU1879 | - | / | ↓ |
| *sbp1* | SSUSC84_1905 | SSU1885 | - | / | / |
| *pili* | SSUSC84_1906 | SSU1886 | - | / | /& |
| *sbp2* | SSUSC84_1907 | SSU1887 | MUN40_10000 | / | / |
| *collagen-binding protein* | SSUSC84_1908 | SSU1888 | - | / | / |
| *gdpp* | SSUSC84_1958 | SSU1940 | MUN40_11065 | / | / |
| *gidA* | SSUSC84_1959 | SSU1941 | MUN40_11070 | / | / |
| *guaB* | SSUSC84_1978 | SSU1960 | MUN40_11245 | / | / |

-: absent in the corresponding genome

/: non-regulated in the corresponding interaction group

&: PIRG

↑:upregulated,↓:downregulated
