# Supplementary figures and images for: Comparative transcriptomic analysis reveal genes involved in the pathogenicity increase of Streptococcus suis epidemic strains
Source: Virulence. 2022 Aug 28;13(1):1455–70. doi: 10.1080/21505594.2022.2116160 (PMC9423846; doi:10.1080/21505594.2022.2116160)

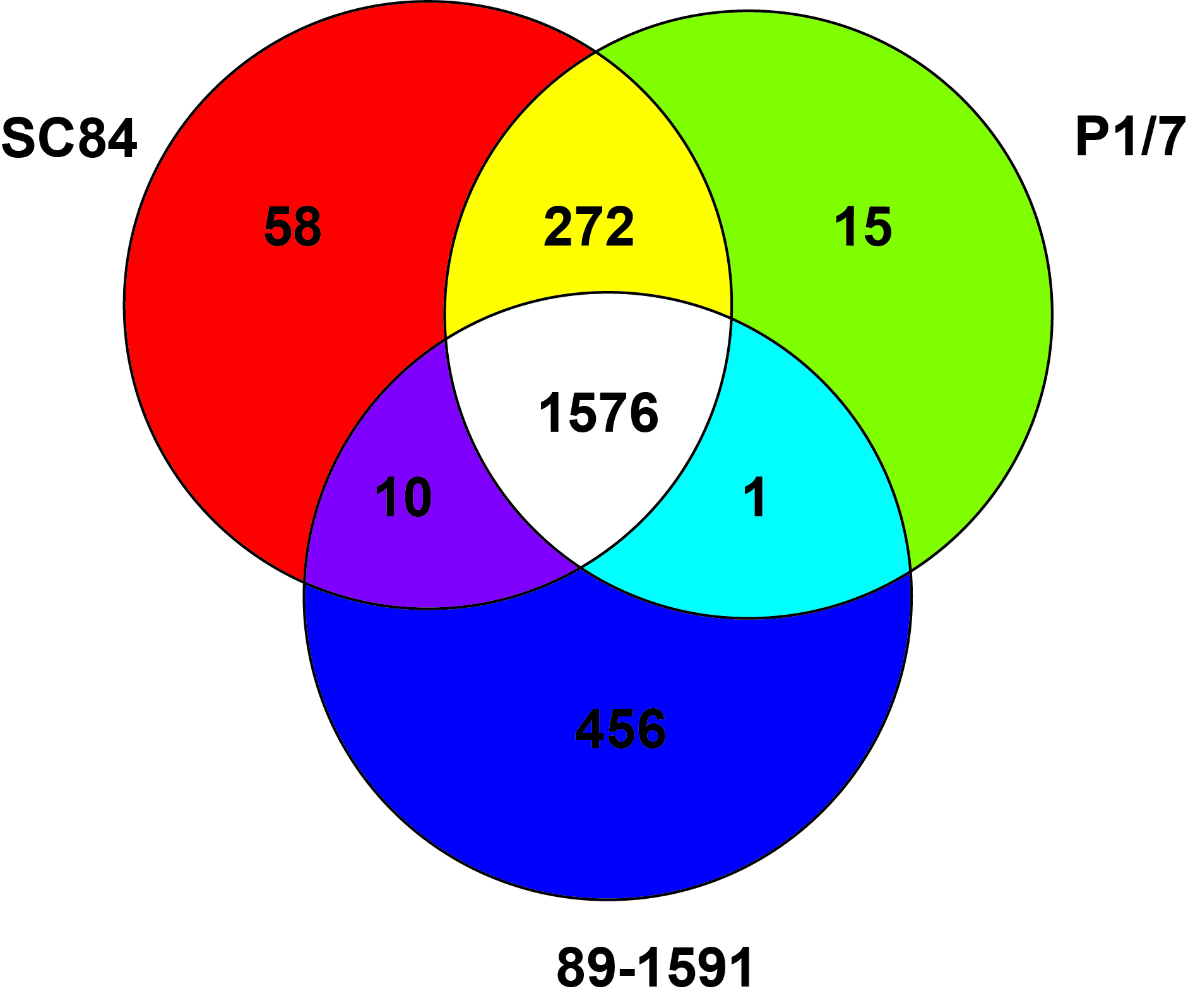

Supplement: Supplemental Material [file KVIR_A_2116160_SM1757.zip › supplementary/Supplemental Figure 1.jpg]

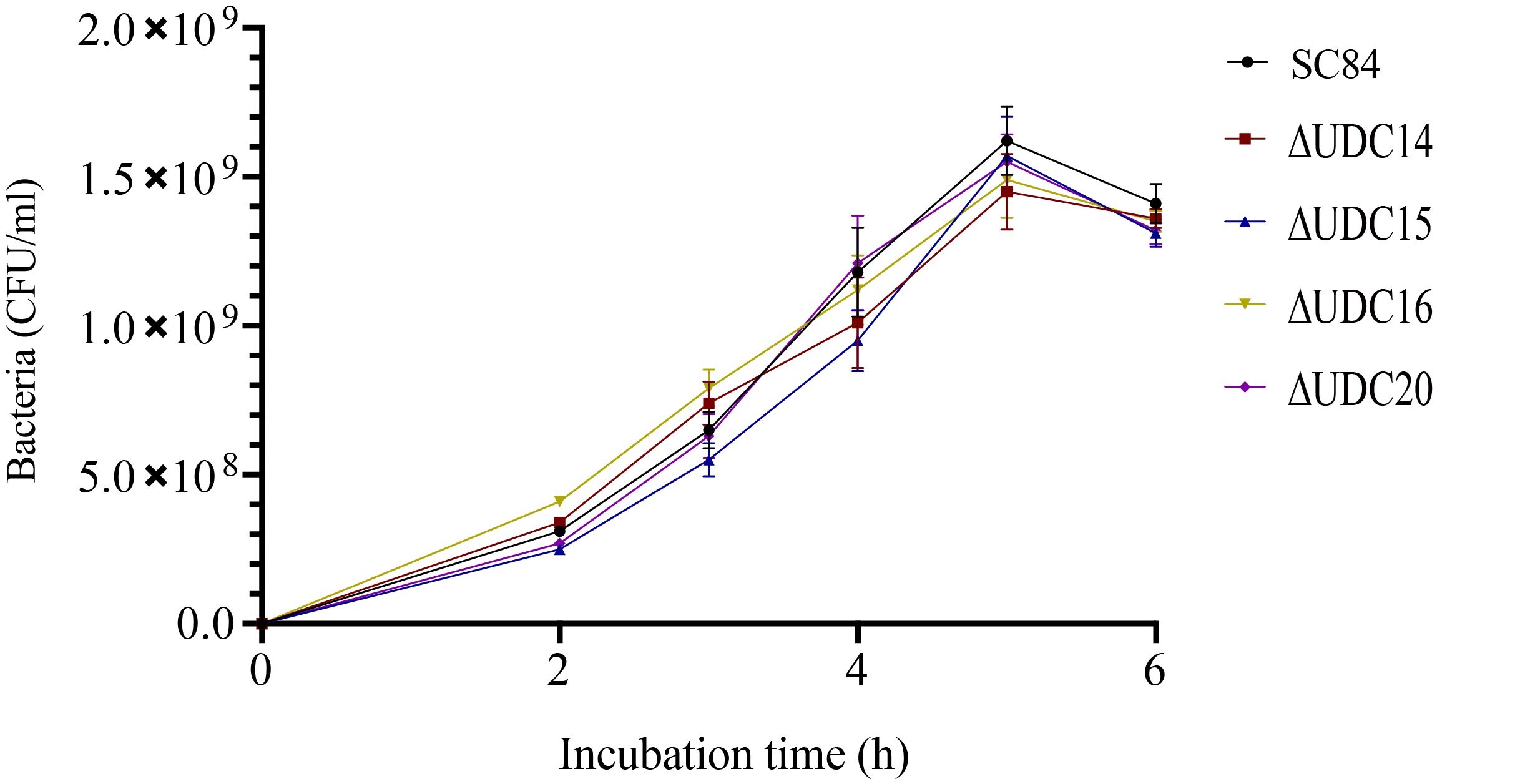

Supplement: Supplemental Material [file KVIR_A_2116160_SM1757.zip › supplementary/Supplemental Figure 2.jpg]
